# Hitting the target: cell signaling pathways modulation by extracellular vesicles

**DOI:** 10.20517/evcna.2024.16

**Published:** 2024-09-24

**Authors:** Giada Cerrotti, Sandra Buratta, Raffaella Latella, Eleonora Calzoni, Gaia Cusumano, Agnese Bertoldi, Serena Porcellati, Carla Emiliani, Lorena Urbanelli

**Affiliations:** ^1^Department of Chemistry, Biology and Biotechnology, University of Perugia, Perugia 06123, Italy.; ^2^Centro di Eccellenza sui Materiali Innovativi Nanostrutturati (CEMIN), University of Perugia, Perugia 06123, Italy.

**Keywords:** Extracellular vesicles, exosomes, microvesicles, Wnt, Notch, Hedgehog, TGF-β, EGFR, Hippo

## Abstract

Extracellular vesicles (EVs) are lipid bilayer-enclosed nanoparticles released outside the cell. EVs have drawn attention not only for their role in cell waste disposal, but also as additional tools for cell-to-cell communication. Their complex contents include not only lipids, but also proteins, nucleic acids (RNA, DNA), and metabolites. A large part of these molecules are involved in mediating or influencing signal transduction in target cells. In multicellular organisms, EVs have been suggested to modulate signals in cells localized either in the neighboring tissue or in distant regions of the body by interacting with the cell surface or by entering the cells via endocytosis or membrane fusion. Most of the EV-modulated cell signaling pathways have drawn considerable attention because they affect morphogenetic signaling pathways, as well as pathways activated by cytokines and growth factors. Therefore, they are implicated in relevant biological processes, such as embryonic development, cancer initiation and spreading, tissue differentiation and repair, and immune response. Furthermore, it has recently emerged that multicellular organisms interact with and receive signals through EVs released by their microbiota as well as by edible plants. This review reports studies investigating EV-mediated signaling in target mammalian cells, with a focus on key pathways for organism development, organ homeostasis, cell differentiation and immune response.

## INTRODUCTION

Extracellular vesicles (EVs) are a heterogeneous family of nanoparticles surrounded by a double-layer lipid membrane. These nanostructures have been isolated from both eukaryotes and prokaryotes, including animals, parasites, plants, and microorganisms. In humans, EVs have been retrieved from all biological fluids analyzed so far: blood, urine, saliva, tears, milk, sweat, as well as cerebrospinal, bronchoalveolar, ascitic, amniotic, synovial, and follicular fluid. Additionally, they are released in the growth medium by cultured cells, thus facilitating the in vitro investigation of their properties in a variety of cell models^[1]^. From a functional point of view, EVs have been initially considered an additional mechanism of elimination of unnecessary or potentially harmful cell content. Although this role has proven to be relevant^[2]^, substantial evidence has indicated that they also represent an additional modality of cell-to-cell communication^[3]^. Indeed, EVs contain molecules, such as proteins, lipids, metabolites, and different types of nucleic acids, that allow them to interact with target cells and play a role in physiological, as well as pathological, processes^[4]^. In this review, we will concentrate our attention on recent studies investigating EV-mediated signaling in target cells, with a focus on pathways modulated in mammalian cells by EVs via the presence of signaling pathway components, such as ligands and receptors.

## EV PROPERTIES

EVs are a heterogeneous family of nanoparticles, and based on their origin, three main types have been defined. Exosomes are small particles (30-120 nm) originating from the inward invagination of late endosomal membranes that produce intraluminal vesicles (ILVs) within the endosome, called for this reason multivesicular bodies (MVBs). ILVs may be either released extracellularly upon exocytosis or delivered to lysosomes for degradation^[5]^. Microvesicles (100-1000 nm), sometimes termed ectosomes, derive from the outward budding of plasma membrane^[6]^. Apoptotic bodies (50-5000 nm) are generated during apoptosis by cell blebbing^[7]^. Despite their easy definition based on their biogenesis, these EVs have overlapping properties in terms of size and density. The current most employed isolation techniques, such as differential centrifugation, possibly followed by density gradient separation, size exclusion chromatography, and polymer precipitation, do not allow separation, for instance, of exosomes from microvesicles^[8]^. Consequently, the current International Society for Extracellular Vesicles (ISEV) guidelines recommend using the terms small EVs, enriched in exosomes, and medium/large EVs, possibly enriched in microvesicles, according to the separation method employed, unless the biogenesis process was specifically investigated^[9,10]^. Additionally, the complexity of the EVs family has increased over the last decade. EVs also include large particles, such as migrasomes (500-3000 nm) released from retraction fibers during cell migration^[11]^, large oncosomes (1-10 µm) released from cancer cells^[12]^, and other types of less characterized nanovesicles^[13]^. In addition, non-vesicular (not surrounded by a lipid bilayer) extracellular particles, such as exomeres (less than 28-50 nm)^[14]^ and supermeres (22-32 nm)^[15]^, have also been described. The number of EV subtypes is still increasing, and their classification could possibly be re-shaped in the next few years by the addition of new members, in synergy with the development and setting up of innovative separation technologies.

The biochemical contents of EVs have shed light on their function. First, EVs contain proteins that are related to their biogenesis process. As for exosomes, biogenesis may either rely on the endosomal sorting complex required for transport (ESCRT) or be ESCRT-independent. ESCRT-dependent biogenesis requires the ESCRT complexes, which are soluble multiprotein complexes, termed ESCRT-I to -IV, recruited to the cytosolic side of the endosome to sort selected proteins into ILVs^[16]^. ESCRT-independent biogenesis has been reported to rely on ceramide produced by sphingolipid metabolism or on tetraspanin oligomerization^[17,18]^. ESCRT components (such as Alix and Tsg101) and tetraspanins usually co-localize with small EVs enriched in exosomes, although they can also be detected in larger EVs^[8]^. As for microvesicles, biogenesis requires the cargo buildup on the cytosolic side of the plasma membrane and its outward protrusion and fission^[19]^. Therefore, biogenesis and release happen almost simultaneously. Enzymes that modulate membrane curvature by acting on phospholipids, such as sphingomyelinases, are involved in this process^[20]^. In addition, the ESCRT machinery is also implicated in microvesicle biogenesis, as the knockdown of ESCRT proteins, such as Alix and Tsg101, affects their formation^[21]^. Other proteins, such as ARF6, Rab and Rho small GTPase family members, are also implicated^[22]^.

The EV biochemical contents are complex. As mentioned above, EVs contain several proteins involved in their biogenesis process, such as ESCRT components and tetraspanins, which are enriched in small EVs but can also be detected in medium/large EVs. In addition, EVs usually contain proteins that are specific to the releasing tissue and/or cell, with many of them involved in signaling processes^[17]^. The EV double-layer membrane is usually enriched in cholesterol and sphingomyelin, resembling that of detergent-resistant microdomains^[4]^, but the presence of bioactive lipids and/or precursors has also been reported^[20]^, thus suggesting that EVs may spread lipid-mediated signaling. Finally, a remarkable feature of EV cargo is related to the presence of nucleic acids. The discovery that EVs contain many types of RNA and DNA has been a breakthrough in the field. EVs were shown to transfer mRNA, as well as miRNA, into recipient cells, thus contributing to modulating gene expression in target cells through horizontal transfer of genetic information^[23]^. More recently, other types of RNA have been reported to be associated with EVs, such as long noncoding RNA^[24]^, circular RNA^[24]^, and transfer RNA^[25]^. The DNA associated with EVs has been reported to be of either genomic or mitochondrial origin^[26]^. A further layer of complexity to EV biochemical contents was added by the discovery of their “protein corona”. When released extracellularly, EVs bind free ligands and other molecules they have an affinity for, such as proteins and nucleic acids. Although these molecules are not loaded into EVs during their biogenesis, they impact their function^[27]^.

EVs have been implicated in a variety of processes, both physiological and pathological, i.e. immune response, cancer biology (including metastasis), tissue homeostasis (including regenerative processes such as angiogenesis and wound healing), and also in degenerative processes such as aging and age-associated disorders^[28]^. However, most details related to the molecular mechanisms underlying the role of EVs in these processes still need to be fully understood.

## EVs AS MEDIATORS OF CELL-TO-CELL COMMUNICATION

Following their release, EVs interact with target cells, either in neighbor tissues or in distant regions of the body. The interaction of EVs with target cells has been extensively investigated and may occur in different ways. In addition, there is evidence that a single cell may release different types of EVs, and it is not possible to exclude that different types of EVs released from the same cell may interact with the same target cell in different manners^[29]^. EVs may deliver their contents to the cytoplasm by direct membrane fusion^[30]^. On the opposite side, EVs in the proximity of a cell may exert their effect by binding to cell surface receptors without requiring cargo transfer into the recipient cell^[31]^. A typical and well-characterized example of this type of interaction without cell entry is the activation of T cells by the MHC class II-antigen complex localized on the EV surface^[20]^. However, the interaction of EVs with receptors on target cells may also lead to EV internalization via endocytosis. The endocytic process may occur via different mechanisms that may be either relatively unspecific, such as macro or micropinocytosis, or receptor-specific, such as clathrin-dependent and clathrin-independent endocytosis^[32]^. Irrespective of the mechanism, endocytosis leads to EV internalization into endosomes. These may either release their contents into the cytoplasm by back fusion with the endosomal membrane or deliver them into lysosomes for degradation and/or recycling. Moreover, there is also evidence that EVs may enter the endosomal system of a target cell and be released extracellularly as intact as they entered^[33]^. Independently from the mechanism of EV-target cell interaction, current evidence implicates EVs in the modulation of several cell signaling pathways required for fundamental biological processes during development, as well as for the communication among tissues and organs in normal or stress conditions. In the next sections, we summarize the role of EVs in mediating morphogenetic signals involved not only in embryo development but also in pathological and physiological processes such as cancer, i.e., Wnt, Hedgehog (Hh), Notch and Eph/ephrins. We also report on the current literature related to the role of EVs in cell signaling pathways mediated by cytokines and growth factors involved in tissue differentiation and proliferation, such as transforming growth factor β (TGF-β) and epidermal growth factor (EGF). Many studies investigated the activation of cell signaling pathway components in target cells and described the activation of protein kinases, such as extracellular signal-regulated kinase (ERK)^[34-37]^, AMP-activated protein kinase (AMPK)^[38,39]^, phosphatidylinositol 3-kinase (PI3K)^[40-44]^, or the modulation of transcription factors function, such as the inhibition^[45]^ or activation^[46]^ of nuclear factor kappa-light-chain-enhancer of activated B cells (NF-B). However, our attention will mostly focus on studies describing the presence of signaling elements in EVs, such as ligands and receptors, or even nucleic acids.

### The role of EVs in morphogenetic signaling pathways

Morphogens are substances involved in determining cell and tissue differentiation, as well as cell fate and proliferation, during embryonic development. A subset of morphogens is released into the extracellular environment and mediates cell-to-cell communication^[47]^. Among morphogens, Wnt and Hh have been extensively studied over the last decades. These secreted morphogens initially represented a challenge since, from a biochemical point of view, they are unable to disperse as soluble factors in the extracellular space because they are post-translationally modified by adding a lipid moiety and associated with the plasma membrane bilayer. A key discovery in solving this puzzle has been the evidence that Wnt and Hh proteins are carried by EVs and that EVs play a key role in transmitting these morphogenetic signals^[48]^. Notch and Eph/ephrin pathways both play a role in a variety of developmental processes, including neurogenesis. Their activation was thought to require physically adjacent cells, but the discovery of Notch and Eph/ephrin components associated with EVs demonstrated that this may not be a mandatory requirement. In the next sections, we are going to summarize how investigations on EVs have added a layer of complexity to the molecular mechanisms involved in these signaling pathways, all mediating fundamental biological processes, and have unraveled EVs as an essential component in the transmission of these signals.

#### The Wnt pathway

Wnt are a family of 19 proteins in vertebrates^[49]^ that act as ligands for several receptors and co-receptors. Among these, the best characterized are the Frizzled family of receptors, which are 7-transmembrane G protein-coupled receptors (GPCRs) localized in the plasma membrane of target cells. Wnt signaling comprises both canonical and non-canonical pathways^[50]^. The canonical Wnt pathway relies on the intracellular activation of β-catenin, which translocates into the nucleus and activates transcription factors of the T-cell factor/lymphoid enhancer factor (TCF/LEF) family, regulating the expression of target genes such as matrix metalloproteinases and c-Myc. It is known as the Wnt/β-catenin pathway and is involved in regulating cell proliferation, including in pathological conditions like cancer^[51]^. The non-canonical Wnt pathways are β-catenin-independent and the two best characterized are the Wnt planar cell polarity and the Wnt/Ca^2+^ pathways, involved in the regulation of cell polarity and migration^[52]^. In addition, the Wnt/receptor tyrosine kinase-like orphan receptor (ROR) pathway also emerged as a non-canonical pathway because of its role in cancer^[53]^.

**Figure 1:**
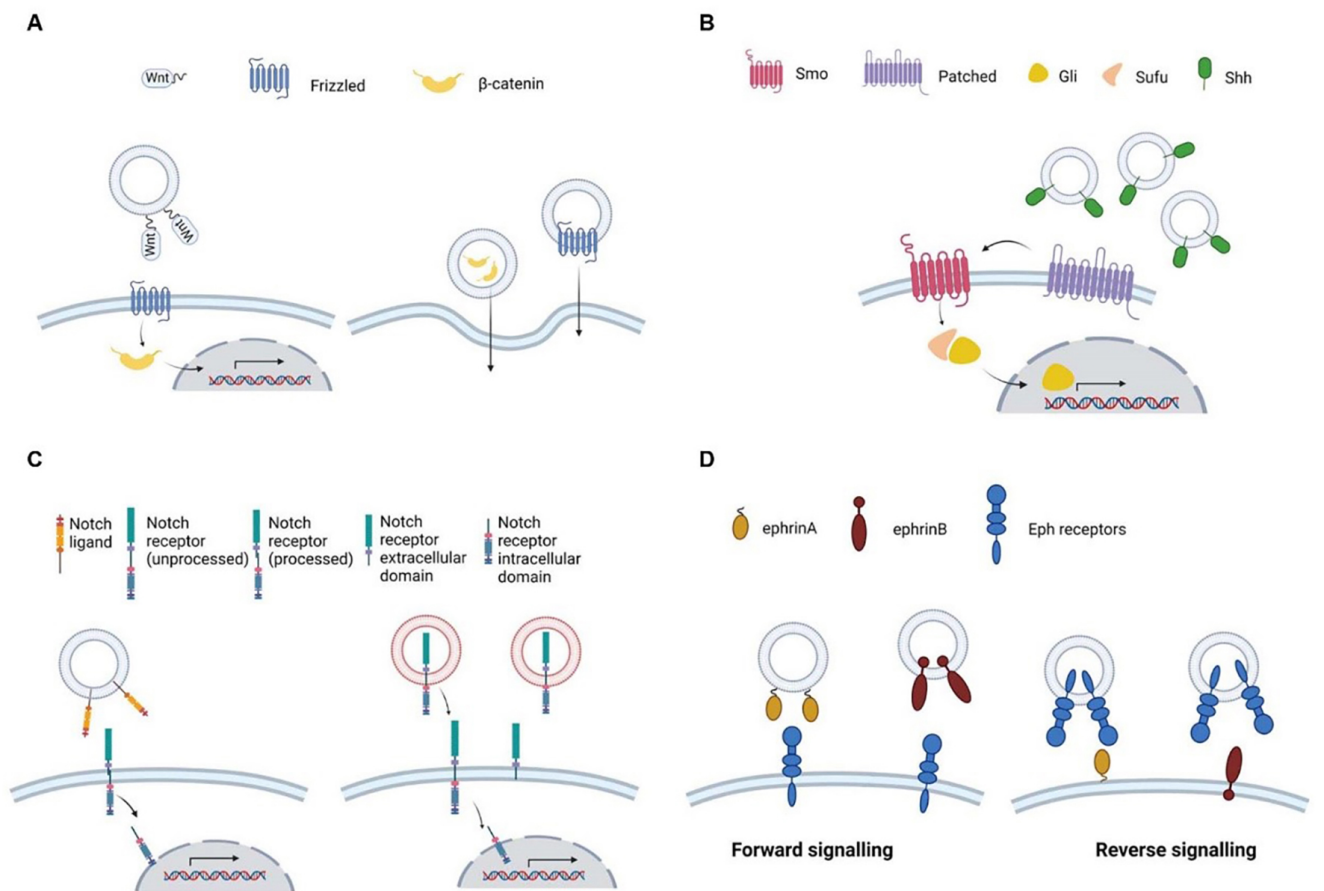
Schematic representation of EV-mediated morphogen signaling. A: Wnt ligands localized on the EV surface may bind to Frizzled receptors on a target cell, activating the canonical Wnt pathway. EVs may also contain β-catenin transcription factor or Frizzled receptor, and deliver them to target cells, thus converting them into cells responsive to Wnt pathway activation. Co-receptors are not shown; B: Shh localized on the EV surface may bind to Patched receptor, removing its inhibitory action on Smo receptor. In turn, activated Smo promotes Gli translocation into the nucleus; C: Notch ligands present in the membrane of EVs can bind to Notch receptors localized on the target cell surface (left panel). The presence of unprocessed Notch receptors on ARMMs can lead to the delivery of Notch receptors to the target cell, making it responsive to Notch signaling (right panel); D: EVs may carry either ephrin ligands (A and B), activating forward signaling in target cells expressing Eph receptors (left panel), or Eph receptors, activating reverse signaling in target cells (right panel). EV: Extracellular vesicles; Smo: Smoothened; ARMMs: arrestin domain-containing protein 1-mediated microvesicles; Sufu: suppressor of fused; Shh: sonic Hedgehog.

The presence of Wnt signaling components in EVs is well documented. In 2010, Chairoungdua *et al*. first reported that CD82 and CD9 expression induces β-catenin export via exosomes, which was blocked by a sphingomyelinase inhibitor, GW4869^[54]^. Gross *et al*. showed that Wnts are secreted in exosomes during *Drosophila* development and in human cells^[55]^. Interestingly, also evenness interrupted (Evi), which is required in *Drosophila* for the secretion of Wnt1, was found to be secreted in exosomes^[55,56]^ and Wnt5a was reported to be localized both in plasma membrane-derived microvesicles as well as in exosomes from macrophages and breast cancer cells^[57]^. Both β-catenin^[58]^ and oncogenic mutant β-catenin^[59]^ were found to be secreted in EVs and able to activate the oncogenic Wnt pathway in target cells. More recently, Frizzed 10 receptor was found to be associated with EVs^[60]^, as well as tyrosine kinase-like orphan receptors ROR1 and ROR2^[61]^. A few of these studies provided not only evidence of association of Wnt signaling pathway components to EVs, but also confirmed their ability to activate Wnt signaling in target cells. Dovrat *et al*. observed that 14-3-3 and β-catenin are secreted via EVs to induce Wnt signaling in HEK target cells^[58]^. Kalra *et al*. reported that oncogenic β-catenin in EVs released by a colon cancer cell line stimulated Wnt signaling in a colon cancer cell line expressing wild-type β-catenin^[59]^. This study was particularly relevant because it showed that EVs may transfer the oncogenic mutated version of a protein to a cell expressing the wild-type protein, leading to the activation of oncogenic signaling^[59]^. This finding underlines the notion that EVs may act as a shuttle, delivering key molecules to target cells lacking the pathway components necessary for signaling activation [Figure 1A]. Due to its relevance in regulating morphogenetic processes such as cell proliferation and cell migration, which are also relevant in cancer biology, the Wnt signaling pathway has been implicated in tumor initiation as well as in tumor progression and metastasis^[62]^. Accordingly, evidence has suggested that EVs carrying Wnt components are involved in different aspects of oncogenesis by activating Wnt signaling in cells undergoing oncogenic transformation as well as in cells localized in the tumor microenvironment, affecting processes such as cell migration and proliferation^[61,63,64]^, tumor progression^[65]^, metastasis^[66]^, angiogenesis^[67]^, and resistance to chemotherapy^[68]^. Besides cancer, EV-mediated Wnt signaling has also been implicated in the modulation of tissue crosstalk. For example, non-pigmented ciliary epithelium cells release EVs targeting primary trabecular meshwork cells to regulate aqueous humor production^[69]^ and EV-mediated Wnt signaling regulates tissue fibrosis via β-catenin in kidney^[70]^ and liver^[71]^.

#### The Hh pathway

The Hh pathway consists of only one gene in *Drosophila* but includes three paralogs in vertebrates, among which Sonic Hedgehog (Shh) is the best characterized^[72]^. It is present in all organisms presenting a bilateral symmetry and is required in the embryo, as its gradient in embryonic cells is necessary to orchestrate differentiation signals^[73]^. Due to its involvement in cell differentiation, it has also been implicated in pathological processes related to abnormal cell differentiation, like cancer^[74]^. As in the case of Wnt, all Hh proteins are lipidated: the N-terminus, which carries the biological activity, is palmitoylated, whereas a cholesterol moiety is added to the C-terminus. Additionally, similar to Wnt, Shh can activate either a canonical or a non-canonical pathway. Shh binds to Patched 1, a 12-pass transmembrane cell surface receptor, which modulates the Smoothened (Smo) receptor, a 7-pass transmembrane cell surface receptor. Smo, in turn, acts on downstream targets, i.e. the Gli transcription factor family members, Gli1, Gli 2, and Gli 3. In the absence of the Shh ligand, Patched 1 represses Smo activity, and consequently downregulates Gli members activation, maintaining them in the cytoplasm, so the transcription of their target genes is repressed. The suppressor of fused (Sufu) is one such negative regulator of Hh signaling. In the presence of the Shh ligand, Patched 1 is addressed to lysosomes for degradation, and Smo is active and leads to nuclear translocation of Gli members and activation of Hh target genes [Figure 1B]. The non-canonical Hh pathway instead refers to the activation of Gli factors independently of the Smo status. A variety of signaling pathways have been reported to be involved in the non-canonical activation of Gli, like MAPK, PI3K/protein kinase B (AKT)/mammalian target of rapamycin (mTOR) and protein kinase C (PKC)^[75]^.

The first evidence that EVs could be involved in mediating the activation of the Hh pathway came from studies investigating the role of the endosomal system in modulating Hh signaling in model organisms. In 2006, Liégeois *et al*. first demonstrated that in *C. elegans*, the vacuolar ATPase, responsible for maintaining an acidic pH in the endo-lysosomal compartment, was required for the secretion of Hh via a multivesicular compartment^[76]^. In 2014, key studies in *Drosophila* reinforced this evidence: Matusek *et al*. showed that ESCRT-positive vesicles transported Hh extracellularly^[77]^, whereas Gradilla *et al*. reported that Hh was localized in vesicles transported via the cytoneme, a specialized type of signaling filopodium^[78]^. These studies calculated that EV-mediated Hh transport accounted for about 50% of signaling activation. However, calculations were mostly based on impaired signaling following the perturbation of ESCRT components, and it is necessary to remember that the ESCRT system has pleiotropic and fundamental functions, so that the complete knockdown of most components is impossible. Soon after these studies were published, studies on vertebrates provided similar evidence. Vyas *et al*. observed that Shh was secreted in two types of EVs with different signaling properties: both Shh-containing vesicles could activate a Gli-luciferase construct, but only the vesicle-type co-expressing integrins could activate endogenous Shh target genes^[79]^. This was a very interesting observation, suggesting that the signaling properties and abilities of EVs may rely on the combinations of proteins on their surface and not on the presence of a single signaling molecule. The same group later reported that oligomerization-defective Hh was not incorporated into EVs because it could not be efficiently internalized into MVBs^[80]^. The evidence that Shh may be released via a specific subpopulation of EVs was further reinforced in another study demonstrating that the human microcephaly gene *CHMP1A* regulates the secretion of Shh into a subpopulation of EVs containing AXL, Rab18, and transmembrane emp24 domain-containing protein 10 (TMED10), called ART vesicle for this reason^[81]^. Recently, the relevance of the partition of Hh not generically in EVs but in specific EV subpopulations has been reinforced by a study also in *Drosophila* showing that the signals for wing imaginal disc development were carried out by EVs released from microvilli, structures that characterize the apical membrane of epithelial cells^[82]^. The authors proposed that EVs released from microvilli at the apical pole of the epithelial cells mediate the long-range effect of the morphogen, whereas those released from the basolateral zone were responsible for Hh short-range activity.

The developmental biology studies demonstrating the presence of Hh in EVs have prompted studies investigating the role of EV-mediated Hh signaling in cancer. Zhao *et al*. reported that Shh localized in EVs released from cancer-associated fibroblasts (CAFs) promoted the proliferation and migration of esophageal squamous carcinoma cells^[83]^. In melanoma, EVs derived from hyaluronan synthase 3-overexpressing cells led to the upregulation of c-Myc through the Indian Hh signaling pathway^[84]^. When Shh was knocked down in a hepatocellular carcinoma cell line, the ability to form tumors in a xenograft model by co-injecting cells and their released EVs was impaired^[85]^. Cervical cancer-derived EVs were shown to promote a pro-angiogenic response in endothelial cells through the transfer of Hh–Gli signaling components^[86]^. An effect on angiogenesis was reported by Zhou *et al*., as EVs released from periodontitis-compromised dental pulp stem cells promoted local angiogenesis by activating the Hh/Gli1 signaling^[87]^. During high-fat exposure, EVs released by human adipose-derived stem cells inhibited lipogenesis via activation of the Hh signaling pathway^[88]^.

#### The Notch pathway

Notch is an evolutionarily conserved pathway that plays a major role in a variety of processes in the context of developmental biology, including cell fate determination during lineage differentiation^[89]^. A distinguishing feature of the Notch pathway is that ligands are localized on the surface of the plasma membrane and bind to transmembrane receptors localized on adjacent cells, so signals are usually transmitted among physically close cells^[90]^. In humans, five Notch ligands have been described: delta-like ligand (DLL) 1, DLL3, DLL4, Jagged-1 (JAG1) and JAG2^[91]^, as well as four Notch receptors (Notch1, 2, 3, and 4). All of them are transmembrane proteins. Notch receptors reach the plasma membrane by the ER-Golgi secretory route. During this process, they are extensively modified by glycosylation (in the ER) and proteolytic cleavage (in the Golgi), producing a heterodimer made of a Notch fragment non-covalently bound to another fragment, a type I transmembrane protein^[92]^. The first Notch fragment is localized extracellularly and interacts with Notch ligands on the adjacent cell. This interaction leads to the proteolytic cleavage of the second fragment in its transmembrane moiety, guiding the release of the intracellular domain, which translocates into the nucleus and acts as a transcription factor. The activation of the Notch receptor upon ligand binding is known as the canonical Notch signaling pathway. However, the Notch pathway can also be initiated by atypical ligands in a non-canonical manner. In this case, intracellular proteases, such as the -secretase, are responsible for Notch receptor cleavage^[93,94]^.

The first evidence that EVs could be involved in mediating Notch signaling was reported by Sheldon *et al*.^[95]^. In this study, authors showed the presence of the Notch ligand DLL4 in EVs released by both endothelial cells and tumor cells overexpressing DLL4. The presence of DLL4 in EVs and its ability to activate Notch signaling in endothelial cells was confirmed in another study^[96]^. Tan *et al*. revealed the presence in EVs of another Notch ligand, JAG1, providing evidence that JAG1-EVs inhibited Notch signaling and, consequently, endothelial cell proliferation and migration^[97]^. The presence of JAG1 was also reported in EVs from fetal dermal mesenchymal stem cells (MSCs). In this case, JAG1-EVs were reported to increase adult dermal fibroblast cell motility and secretion, accelerating cutaneous wound healing, via the activation of Notch signaling^[98]^. Besides the canonical activation of Notch signals, there is also evidence of EV-mediated non-canonical Notch signal activation^[99]^. Arrestin domain-containing protein 1-mediated microvesicles (ARMMs) originating from the outward budding of the plasma membrane were demonstrated to contain active Notch2 receptors. When ARMMs were delivered to recipient cells, Notch2 receptor was activated by proteolytic cleavage, leading to Notch-dependent gene expression in EV-recipient cells. This report is of particular interest because, in the case of Wnt and Hh signaling, the evidence of the association of signaling components with EVs explained how these membrane-anchored proteins such as Wnt and Hh could diffuse extracellularly, whereas in the case of Notch signaling, the presence of Notch2 receptor in ARMMs indicated an additional mechanism for transmitting Notch signals, i.e. at a distance via EVs, without requiring direct cell-to-cell contact. In summary, current evidence indicates that EVs may be responsible either for the activation of canonical Notch signaling at a distance via the presence of Notch ligands in EVs, or for the non-canonical activation of Notch signaling via the uptake of Notch receptors present in EVs [Figure 1C].

Notch signaling is involved in cancer development and many reports have underlined that EV-associated Notch components may play a relevant role in this process. Initially, the presence of Notch ligands or receptors in EVs was not directly detected, but Boelens *et al*. observed that vesicles released from stromal cells induced Notch3 activation in breast cancer cells^[100]^, whereas Wang *et al*. reported that breast cancer cells release EVs promoting breast cancer cell drug resistance via Notch1 activation^[101]^. Consistently, Yang *et al*. showed that EVs released by endothelial cells favored osteosarcoma cell stemness by activating the Notch signaling pathway^[102]^. More recently, Giannandrea *et al*. investigated EV-associated Notch2 in the tumor microenvironment, revealing that multiple myeloma cells can transfer Notch2 to distant cells via EVs, increasing Notch signaling in recipient cells and stimulating the pro-tumorigenic behavior of endothelial cells and osteoclasts^[103]^.

#### Eph/ephrin pathway

Eph receptors and ephrin (Eph receptor-interacting protein) ligands are fundamental players in many processes, including axon guidance, cell adhesion/repulsion, migration, and proliferation. As in the case of Notch, this pathway is characterized by the fact that both Eph receptors and ephrin ligands are plasma membrane proteins, so their activation occurs via direct cell-to-cell contact^[104]^. Eph receptors are divided into A and B classes (10 and 6 members in mammals, respectively). EphA receptors bind to all ephrin-A ligands, whereas EphB receptors bind to ephrin-B ligands. EphA and EphB receptors are both single-pass transmembrane proteins with a complex extracellular ephrin-binding domain and intracellular tyrosine kinase activity. Ephrin-A ligands are anchored to the plasma membrane through a glycosylphosphatidylinositol moiety and ephrin-B ligands are single-pass transmembrane proteins with a small cytoplasmic domain^[105]^. Upon Eph/ephrin binding, a peculiar bidirectional signaling is activated, termed forward signaling in Eph receptor-expressing cells and reverse signaling in ephrin ligand-expressing cells^[106]^. Consequently, Eph/ephrin interaction was initially thought to require cell-to-cell contact and be limited to short-range signaling. However, EVs were demonstrated to contain ephrin ligands (B1, B2 and A2) and Eph receptors^[107,108]^. In 2016, Gong *et al*. demonstrated that EVs containing Eph type B2 (EphB2) receptors induced ephrin-B1 reverse signaling, causing neuronal axon repulsion^[109]^. Small EVs released from senescent cells contained Eph type A2 (EphA2) receptors and promoted proliferation via EphA2/ephrin-A1 reverse signaling^[110]^. Sato *et al*. reported that EphB2 receptors carried on EVs activated reverse signaling, inducing angiogenesis^[111]^ [Figure 1D]. The activation of ephrin-Eph forward signaling by EVs was also recently described, as the ephrin-A1 ligand carried by EVs released from metastatic breast cancer cells was shown to activate AMPK^[112]^. Altogether, these findings indicated that ephrin-Eph bidirectional signals may occur not only at a short distance via cell-to-cell contact, but also at a long distance via EVs. The specific biochemical features and functional roles of the two different types of Eph/ephrin signal transmission are unknown.

### Pathways activated by cytokines and growth factors

Cytokines are involved in cell signaling, mostly, but not exclusively, as immunomodulating agents^[113]^. Although most cytokines were reported to be secreted via the classical ER/Golgi secretion route, the presence of cytokines in EVs, such as TGF-β, suggested that this may be an alternative secretory route^[114]^, with EV-associated cytokines participating in the modulation of cytokine-mediated signaling in addition to their soluble forms. Growth factors, such as EGF, are also secreted via the classical ER/Golgi secretion route^[115]^, but EGF was also found to be related to EVs^[116]^, indicating that EV-associated growth factors may participate in the modulation of cell signaling. In this section, we summarize our current knowledge of the role of TGF-β and EGF signaling components associated with EVs, to shed light on the role of EVs as additional carriers of these signaling molecules, usually considered to be released only by the classical ER/Golgi secretory route.

#### TGF-β pathway

Many studies have investigated the role of EVs in mediating TGF-β signaling. TGF-β is a cytokine with several functions in adult organisms as well as in the developing embryo. It regulates cell growth and differentiation, showing mostly immunomodulatory and wound-healing properties^[117]^. TGF-β is released in the extracellular space in an inactive form, whose activation involves an unusual mechanism. Indeed, TGF-β is inactive because of its interaction with the latency-associated peptide (LAP). It becomes active when it dissociates from LAP as a result of mechanical interaction between LAP and integrins on the cell surface. Following this, free TGF-β binds to its receptor on the target cell plasma membrane. TGF-β receptors are of either type I (TGFβRI, also termed TGFBR1) or type II (TGFβRII, also termed TGFBR2). Following ligand binding to TGFBR2, TGFBR2 and TGFBR1 oligomerize and become active kinases^[118]^. Remarkably, co-receptors also have great relevance; for example, the TGFβRIII co-receptor, also known as betaglycan, presents TGF-β ligands to TGFBR2 and TGFBR1 receptors in order to activate downstream signaling. TGFBR1 kinase substrates are the transcription factors of the Smad family members 2 and 3 (Smad2 and Smad3), which, upon phosphorylation, oligomerize with Smad4, accumulate in the nucleus and regulate gene expression^[119]^. In addition to this canonical pathway, non-canonical pathways have been reported to play a role in TGF-β signaling, as the kinase activity of TGFBR receptors may regulate the activity of downstream targets other than Smads, namely Rho, PI3K, and MAPK^[120]^.

**Figure 2:**
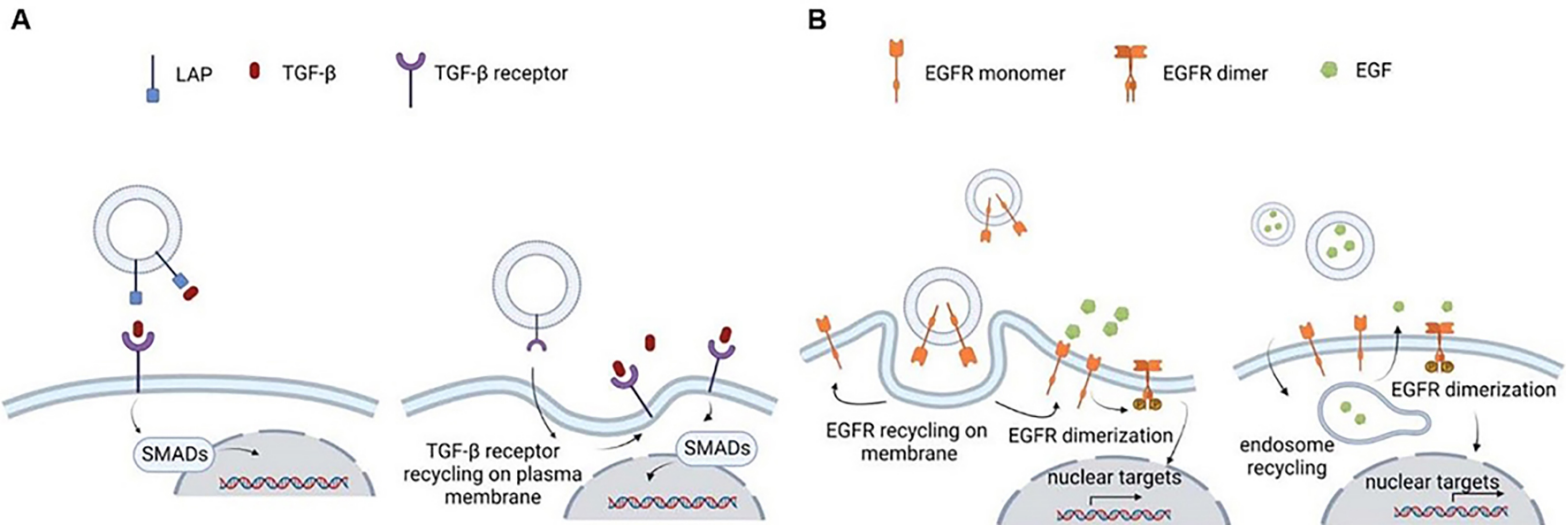
Schematic representation of EV-mediated TGF-β and EGF signaling. A: TGF-β is inactive because of its interaction with LAP. When it dissociates from LAP upon mechanical interaction between LAP and integrins on the membrane, EVs containing TGF-β influence biological processes by binding to TGF-β receptors on the cell surface, whose kinase substrates are the transcription factors of the Smad family. EVs may carry TGF-β type II receptors that are transferred to cells devoid of them, resulting in the activation of TGF-β signaling; B: EGF receptor family members are present on the EV surface. They can be transferred via EVs to cells lacking them, leading to the activation of downstream signaling pathways. EVs may also contain EGFR ligands, leading to the activation of downstream signaling pathways in cells already expressing EGFRs. EV: Extracellular vesicles; TGF-β: transforming growth factor β; EGF: epidermal growth factor; LAP: latency-associated peptide; EGFR: EGF receptor.

Several components of the TGF-β signaling pathway were reported to be associated with EVs [Figure 2A]. Initial studies provided evidence of an immunosuppressive role of TGF-β localized on vesicles. In 2009, Xiang *et al*. reported the presence of prostaglandin E2 (PGE2) and TGF-β in tumor exosomes, both mediating *in vivo* tumor progression via the differentiation of myeloid-derived suppressor cells^[121]^. Xie *et al*. showed that tumor apoptotic bodies inhibit cytotoxic T lymphocyte responses via membrane-bound TGF-β^[122]^. Additional evidence of the immunosuppressive function of vesicular TGF-β toward T regulatory cells (Treg) was observed for EVs isolated from malignant pleural effusions^[123]^. EVs from colorectal cancer cells were enriched in TGF-β and induced a Treg-like phenotype in T cells via TGF-β/Smad signaling^[124]^. Microvesicles in sera from patients with acute myeloid leukemia^[125]^ and from hypoxic tumor cells were shown to inhibit natural killer (NK) cells, an effect dependent on vesicular TGF-β transfer^[126]^. More recently, Ludwig *et al*. reported that head and neck squamous cell carcinoma cells released EVs carrying TGF-β that reprogramed macrophages toward a pro-angiogenic phenotype^[127]^, whereas Fu *et al*. observed that meningioma-associated M2 macrophages released EVs promoting tumorigenesis through the TGF-β pathway^[128]^. The association of TGF-β-enriched EVs with higher immunosuppression was so robust that the suppression of TGF-β expression in cancer cells was used as a strategy to increase the efficacy of tumor exosome-based vaccines^[129]^. Moreover, the level of TGF-β in circulating EVs was used to identify patients that would benefit from immune checkpoint inhibitor therapy in non-small cell lung cancer, as patients with higher levels of EV-TGF-β were considered immunosuppressed and less sensitive to the therapy^[130]^. Beyond tumor immunosuppression, EVs enriched in TGF-β also influenced other pathological processes. Circulating EVs from human immunodeficiency virus (HIV) patients affected by pulmonary hypertension contained higher levels of TGF-β. These EVs enriched in TGF-β1 originated from HIV-infected macrophages differentiated from monocytes and were linked to the pulmonary endothelium damage and smooth muscle hyperplasia that characterize pulmonary hypertension^[131]^.

In the context of the tumor microenvironment, the differentiation into fibroblasts was affected by vesicular TGF-β. Webber *et al*. showed that TGF-β present in EVs was able to activate Smad-dependent signaling, inducing the differentiation of fibroblasts into myofibroblasts^[132]^. From a biochemical point of view, this work is of great interest because vesicular TGF-β was associated with betaglycan and present in its latent form, i.e., complexed with LAP. In addition, it induced the production of higher levels of fibroblast growth factor-2 (FGF-2) by fibroblasts, as compared to soluble TGF-β. The same authors later reported that vesicular TGF-β1 was able to support fibroblast differentiation into a myofibroblast phenotype that could support angiogenesis and accelerate tumor growth, whereas soluble TGF-1 differentiated myofibroblasts that could neither support angiogenesis nor promote tumor growth^[133]^. This evidence that soluble and vesicular TGF-β could co-exist and mediate different biological effects is of great relevance, as it underlines the notion that the release of TGF-β both as a soluble and vesicular protein could be an additional mechanism to finely tune the same cell signaling pathway. A possible molecular mechanism explaining the signaling peculiarities of vesicular *vs.* soluble TGF-β1 was proposed by Shelke *et al*^[134]^. This study identified TGF-β1 on mast cell EVs, discovering that EVs containing TGF-β1 were taken up by MSCs and ended up in the endosomal compartments. This resulted in the prolonged activation of TGF-β1 signaling as compared to soluble TGF-β1. The differentiation of fibroblasts into CAFs by vesicular TGF-β transfer was also reported for EVs released by bladder cancer cells^[135]^, whereas EVs released by gastric cancer cells induced the differentiation of MSCs into CAFs by activation of TGF-β canonical signaling^[136]^. Osteosarcoma EVs containing TGF-β prompted an inflammatory phenotype in MSCs, which became able to promote tumor growth after this treatment^[137]^.

The epithelial-mesenchymal transition (EMT) is another tumor-associated process in which vesicular TGF-β has been involved. TGF-β is a well-characterized inducer of EMT in a variety of cell models^[138]^. EVs carrying TGF-β derived from hepatocellular carcinoma and pancreatic cancer cells could mediate EMT through the TGF-β/Smad signaling pathway^[139,140]^. Mast cell-derived EVs mediated the induction of EMT in A549 airway epithelial cells because they not only carried TGF-β1^[127]^, but also induced TGF-β receptor expression in target cells^[141]^. EVs released from MSCs induced EMT in lung cancer cells, and when TGF-β1 expression was abrogated, EV-mediated EMT in lung cancer cells was also prevented, demonstrating that the effect was mediated by vesicular TGF-β^[142]^. Tan *et al*. provided evidence that resistance to adriamycin in breast cancer cells was associated with the release of EVs characterized by a higher content of TGF-β1^[143]^.

Although many studies on vesicular TGF-β have focused their attention on cancer and cancer-related processes, TGF-β also exerts an important role in processes leading to either tissue regeneration or fibrosis. Kidney tubular epithelial cells in hypoxia conditions produced vesicles that induced fibroblasts to initiate tissue repair, a mechanism dependent on vesicular TGF-β1 mRNA^[144]^. In a diabetic nephropathy model, kidney-infiltrating macrophages released EVs, inducing activation and proliferation of mesangial cells via their vesicular TGF-β1 mRNA^[145]^. In tenocytes, TGF-β1-containing vesicles isolated from bone marrow (BM) MSCs promoted not only proliferation and migration, but also fibrotic activity^[146]^. A positive effect in preventing the apoptosis associated with stroke-induced damage was reported for EVs released from hypoxia-preconditioned microglia and was mediated by TGF-sβ/Smad2/3 signaling^[147]^. The regrowth of neurons in spinal cord injured rats was ameliorated by EVs released by MSCs carrying TGF-β and able to upregulate Smad6 transcription factor in neural stem cells^[148]^.

In addition to ligands, a few studies also described the presence of other components of the TGF-β signaling pathway in EVs. Languino *et al*. reported that stromal fibroblasts isolated from patients with squamous cell carcinoma carried the TGFβ type II receptor^[149]^. This could be transferred via EVs to cancer cells lacking the receptor, resulting in the activation of TGF-β signaling in cells that required this vesicular transfer^[149]^. Gautheron *et al*. observed that EVs released from BM-MSCs contained the TGF-β downstream transcription factor Smad2, which played a pivotal role in the maintenance of hematopoietic stem cells in murine models^[150]^.

#### EGF pathway

The role of EVs in mediating the EGF signaling pathway has been extensively investigated^[116]^, with most studies focused on the presence of EGF receptor (EGFR) in EVs. The EGF signaling pathway involves a family of four receptors: EGFR, also known as HER1 or ERBB1, HER2/ERBB2, HER3/ERBB3, and HER4/ERBB4. These four receptors can form either homodimers or heterodimers. They are all single-pass membrane proteins with an intracellular domain with tyrosine kinase activity, although HER3/ERBB3 is catalytically defective^[151]^. Ligands for EGFR, like EGF, TNF-α, TGF-β, amphiregulin (AREG) and epiregulin, can bind both EGFR homo- and heterodimers. HER3 and HER4 can also bind neuregulins, EGF-related signaling proteins mediating functions in both neuronal and non-neural systems. There are no known ligands for HER2/ERBB2 homodimers^[152]^. Following EGFR homo- or hetero-dimerization upon ligand binding, the intracellular domain tyrosine kinase activity is activated, leading to monomer cross-phosphorylation. Several adaptors can bind phosphorylated residues, triggering cascades that ultimately activate MAPK, PI3K or even signal transducer and activator of transcription 3 (STAT3)^[153]^.

The presence of EGFR in EVs was first described in 2007 by Al-Nedawi *et al*. This study provided evidence that glioma cells expressing the truncated form of EGFR, known as EGFRvIII, can transfer it via EVs to cells lacking this form, leading to the activation of downstream signaling pathways^[154]^. This paper was of great importance because it demonstrated that EVs could biologically mediate the transfer of oncogenic properties to cells lacking oncogenic mutations. Additionally, the truncated EGFRvIII form was also retrieved in circulating EVs from glioblastoma patients^[155]^, reinforcing the evidence of an *in vivo* pathological relevance of oncogenic proteins in EVs as well as the translational importance of EVs as an *in vivo* source of biomarkers. Sanderson *et al*. showed that EVs released by a keratinocyte cell line contained not only the transmembrane form of EGFR but also a soluble form resulting from the cleavage of its extracellular domain by metalloproteases^[156]^. Furthermore, the packaging of EGFR into EVs was stimulated by EGF binding to its receptor, thus shedding light on a mechanism of signal amplification mediated by EVs. Adding a further layer of complexity to the EGFR signaling pathway, EV-associated EGFR was shown to be transported directly into the nucleus, despite the absence of a nuclear localization signal, leading to the direct phosphorylation of proteins important for DNA replication, such as proliferating cell nuclear antigen (PCNA)^[157]^. Many other studies have investigated EV-mediated EGF signaling components in cancer cell models. Functional EGFR was detected in EVs released by several cancer cell lines, such as A431, A549, and DLD-1. These EVs were taken up by endothelial cells showing angiogenic properties, including the ability to prompt vascular endothelial growth factor (VEGF) expression^[158]^. EVs released by cancer cell lines such as A549, HepG2, MCF10A, and MCF-7 prolonged monocyte survival, prompting the development of tumor-associated macrophages (TAM) via the transfer of phosphorylated EGFR, thus promoting an inflammatory microenvironment^[159]^. Transfer of EGFR to macrophages by tumor-derived EVs impaired innate antiviral immunity^[160]^. Lung cancer EVs containing a mutated form of EGFR (EGFR E746-A750) stimulated immunosuppressive activity in dendritic cells (DCs)^[161]^. EVs from cancer cells overexpressing EGFR packaged EGFR in EVs and prompted EMT via an EGFR-dependent pathway^[162]^. EVs from gastric cancer cells containing EGFR deliver it to liver cells, prompting metastasis^[163]^. Tumor-associated viruses such as the Epstein–Barr virus (EBV) provided evidence that EVs released by EBV-infected cells packaged high levels of EGFR, suggesting an effect of EBV infection on the proliferation of neighboring cells via EVs^[164]^. Most of these studies did not specifically investigate the orientation of the tyrosine kinase domain. Depending on the directionality of the tyrosine kinase domain of the receptor-ligand complex, that orientation could differ based on EV biogenesis and uptake route.

In addition to the presence of receptors, a more limited number of studies have reported the presence of ligands in EVs [Figure 2B]. EVs released by breast and colorectal cancer cells carry the EGFR ligands AREG, transforming growth factor α (TGF-α), and heparin-binding EGF-like growth factor (HB-EGF)^[165]^. The presence of AREG was also found in EVs from chronic myeloid leukemia cells and patients’ blood. These EVs were able to activate EGFR signaling in stromal cells, indicating their relevant role in the crosstalk between leukemic and stromal cells, which promotes cancer cell proliferation^[166]^. The presence of AREG in vesicles isolated from non-small-cell lung cancer^[167]^ and multiple myeloma^[168]^ cells was also described, providing evidence that these EVs induced osteoclastogenesis, a process involved in bone metastasis, through the activation of the EGFR pathway. Another ligand, epiregulin, was retrieved in EVs isolated from salivary adenoid cystic carcinoma cells^[169]^. Interestingly, EGFR family ligands were also found in exomeres^[170]^. These are very small nanoparticles (less than 50 nm in diameter) that are not surrounded by a lipid bilayer and contain several metabolic enzymes. Their function remains elusive, but exomeres from a Madin-Darby canine kidney (MDCK) cancer cell line overexpressing AREG presented functionally active AREG that, like the AREG included in small EVs, could elicit prolonged EGFR downstream signaling in recipient cells, effectively enhancing the growth of colonic tumor organoids^[170]^.

#### VEGF pathway

VEGF-A, usually termed VEGF, plays a role in regulating angiogenesis via its binding to VEGF receptors (VEGFR) 1 and 2. It exists in multiple isoforms resulting from alternative exon splicing^[171]^. Few studies have reported the release of VEGF upon treatment with EVs containing EGFR^[151]^ or released by human adipose-derived stem cells^[172]^. A pro-angiogenesis effect was reported for EVs isolated from ovarian cancer cells that increased the expression of VEGFR, leading to higher levels of endothelial cell migration and angiogenesis^[173]^. However, an opposite effect was observed in another study for EVs released by MSCs, which downregulated VEGF expression and angiogenesis in breast cancer cells^[174]^. Some investigations have provided evidence of the direct presence of VEGF signaling components in EVs. In 2006, Taraboletti *et al*. reported that relevant amounts of VEGF but not FGF-2 were detectable in EVs^[175]^. In 2017, Feng *et al*. observed that EVs could activate VEGFRs and tumor angiogenesis through a unique 90 kDa form of VEGF (VEGF90K)^[176]^. Glioblastoma stem cells release EVs containing VEGF-A that have angiogenic effects on endothelial cells^[177]^. Pancreatic adenocarcinoma cells release EVs with an elevated presence of VEGF-C^[178]^. These reports indicate that EVs may play a relevant role in the processes related to angiogenesis in tumors, but more detailed studies are needed to address what cell type and condition could be involved in mediating specific pro- or anti-angiogenic effects.

#### Brain-derived neurotrophic factor pathway

Brain-derived neurotrophic factor (BDNF) plays a fundamental role in brain development and maintenance^[179]^. The human *BDNF* gene is transcribed from nine alternative promoters, translated into the same pre-pro-BDNF and cleaved into the precursor pro-BDNF^[180]^. It binds with high affinity to the tropomyosin receptor kinase B (TrkB) receptor, which activates signaling pathways modulating several neural functions via the activation of downstream effectors such as phospholipase C-, PI3K/mTOR, and MAPK/ERK. Recent investigations have shown that plasma EVs contain BDNF^[181]^ and the level and biochemical properties of BDNF are potential biomarkers for conditions such as walking speed decline in older adults^[182]^ and disturbance-related motor symptoms in patients with Parkinson’s disease^[183]^. In addition, Solana-Balaguer *et al*. found BDNF in EVs derived from neuronal cultures, showing that neuron-derived EVs promote spine formation and activate BDNF-TrkB signaling in recipient neurons, suggesting a potential role of neuron-derived EVs in neuromodulation and as a possible therapeutic tool to fight neurodegeneration^[184]^.

### Intracellular pathways

In the previous sections, we have focused our attention on the activation of cell signaling by EVs containing pathway components such as ligands and receptors. However, there is recent evidence that EV administration may lead to the activation of a cell signaling pathway that does not require a specific cell-surface receptor because it relies on multiple receptors usually activating other pathways, such as Hippo, or involves receptors localized not only on the plasma membrane but also on endosomes, such as the pattern recognition receptor (PRR) pathway. PRRs are key players in innate immune responses, crucial for the elimination of infectious agents^[185]^. They recognize either pathogen-associated molecular patterns (PAMPs) originating from microbes and other external sources^[186]^, or damage-associated molecular patterns (DAMPs) originating from damaged cells^[187]^. This evidence indicates that EVs may exploit complex ways to hit their cellular targets.

#### The Hippo pathway

The Hippo signaling pathway is evolutionarily conserved and plays an important role in cell proliferation and differentiation, as well as in embryogenesis, tissue development, and organ growth^[188]^. It is a peculiar pathway due to its lack of specific cell-surface receptors, but it is activated by several receptors that are usually able to activate other pathways, such as integrins, tyrosine kinases, and GPCRs^[189]^. The Hippo pathway includes four kinases involved in a phosphorylation cascade, with macrophage stimulating 1/2 (MST1/2)/ salvador homolog 1 (SAV1) interaction, leading to MOB1 phosphorylation, and with MOB1, in turn, phosphorylating large tumor suppressor kinase (LATS) 1/2. When the cascade is active, Yes-associated protein (YAP)/transcription activator with PDZ binding motif (TAZ) remains in the cytoplasm. When the cascade is inactive, YAP/TAZ is not phosphorylated and translocates to the nucleus where, upon dimerization with DNA-binding factors of the transcriptional enhanced associated domain (TEAD) family, it regulates the expression of downstream genes, most of which are involved in cellular mechano-responses^[190]^.

**Figure 3:**
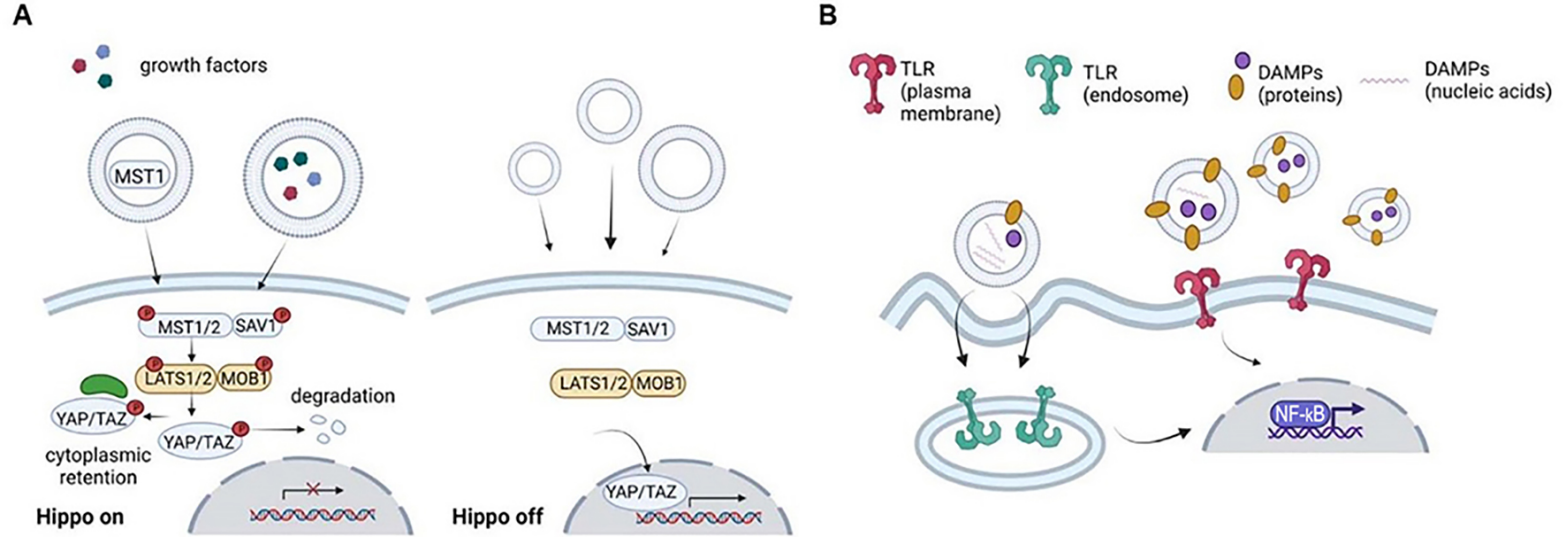
Schematic representation of EV-mediated Hippo and TLRs signaling. A: The Hippo pathway lacks a specific cell-surface receptor and is activated by receptors belonging to other pathways. When the Hippo is on, YAP/TAZ is unable to translocate into the nucleus; when the Hippo is off, YAP/TAZ translocates into the nucleus, activating transcription. EVs may lead to either YAP/TAZ retention/degradation in the cytoplasm or the activation of YAP/TAZ-mediated transcription; B: EVs are vehicles for horizontal dissemination of DAMPs and modulate the immune response via PRRs. TLRs localized in the plasma membrane are activated by DAMPs carried by EVs and TLRs localized in the cytoplasm by EV-associated nucleic acids, in both cases leading to the transcription of NF-B target genes. EV: Extracellular vesicles; TLRs: toll-like receptors; YAP: yes-associated protein; TAZ: transcription activator with PDZ binding motif; DAMPs: damage-associated molecular patterns; PRRs: pattern recognition receptors; NF-B: nuclear factor kappa-light-chain-enhancer of activated B cells.

In 2018, Hu *et al*. demonstrated that EVs from cardiac endothelial cells contain (MST1) kinase and transfer it to cardiomyocytes, leading to cardiomyopathy worsening^[191]^. On the other hand, EVs secreted from BM-MSCs were reported to possess cardioprotective effects, as cardiomyocyte hypertrophy was ameliorated by EVs via downregulation of YAP phosphorylation and upregulation of TAZ, which attenuated the Hippo pathway^[192]^. Several studies suggested that modulation of Hippo signaling by EVs may contribute to alleviating fibrotic processes and promoting cell proliferation, prompting tissue regeneration. In a model of renal fibrosis induced by mechanical stress and characterized by YAP nuclear activation, EVs from human umbilical cord MSCs promoted YAP nucleocytoplasmic shuttling, attenuating renal fibrosis^[193]^. EVs from the human umbilical cord MSCs improved ovarian function in premature ovarian insufficiency, ameliorating the reproductive outcomes in mouse models *in vivo* and the proliferation of granulosa cells in vitro, via nuclear translocation of YAP/TAZ^[194]^. Regenerative properties via the activation of the Hippo pathway were also observed by Wang *et al*., using EVs released by BM-MSCs to treat models of temporomandibular joint osteoarthritis and obtain an improvement in cartilage reconstruction^[195]^. On the other hand, EVs released from astrocytes stimulated neurite elongation and recovery of rats with spinal cord injury via Hippo pathway activation, as measured by higher Mps one binder kinase activator 1A (MOB1A) expression in target cells and reduced YAP levels^[196]^ [Figure 3A].

Dysregulation of the Hippo signaling pathway was shown to be involved in cancer, promoting the migration, invasion, and malignancy of tumor cells^[197]^. YAP/TAZ are commonly identified as oncogenes^[198]^ and MST1/2 and LATS1/2 as tumor suppressors^[199,200]^, although recent evidence suggests that LATS1/2 kinases may also act as oncogenes^[197]^. Accordingly, several studies have investigated the involvement of EVs in modulating the Hippo signaling pathway in cancer. Glioblastoma cells release EVs containing a 120-kDa isoform of VEGF, VEGF-C, that induces the nuclear translocation of TAZ in endothelial cells, stimulating their viability, migration, and tubulation^[201]^. EVs released by MSC-differentiated adipocytes promoted breast cancer cell proliferation and migration via nuclear translocation of YAP/TAZ^[202]^. In cervical cancer, EVs isolated from highly metastatic cells promoted metastasis via YAP-suppressed nuclear localization, leading to F-actin depolymerization^[203]^. In the tumor microenvironment, EVs released by CAFs promoted colorectal cancer progression via MOB1A degradation, leading to Hippo pathway inhibition^[204]^. Gemcitabine resistance of pancreatic cells was promoted by EVs released in hypoxia conditions via the transfer of the long noncoding RNA ROR, which inhibited the activation of the Hippo pathway^[205]^.

#### PRR pathways

PRRs are a complex family and include Toll-like receptors (TLRs)^[186]^, nucleotide-binding oligomerization domain (NOD)-like receptors^[206]^, retinoic acid-inducible gene I (RIG-I)-like receptors (RLRs)^[207]^, the intracellular DNA sensor cyclic GMP-AMP synthase (cGAS)^[208]^, and C-type lectin receptors^[209]^. TLRs (10 members in humans, termed TLR1 to 10) are the most extensively investigated up to now and are expressed not only in innate immune cells, but also in epithelial cells and fibroblasts^[210]^. They are single-spanning transmembrane proteins and recognize PAMPs or DAMPs as heterodimers^[186]^. Their subcellular localization is very relevant for their function, as TLRs may be either localized in the plasma membrane or in the endosomal compartment, and the type of pattern recognized changes according to their localization. Indeed, TLRs localized in the plasma membrane, such as TLR2, 4 and 10, recognize microbial membrane components, i.e., lipids, proteins, and lipoproteins, whereas endosomal TRLs, such as TLR3, 7, 8, and 9, recognize nucleic acids of internalized microbes and viruses, as well as self-derived nucleic acids in specific conditions^[211]^. Following molecular pattern binding, several adaptor proteins, such as myeloid differentiation primary response 88 (MyD88), can be recruited by TLRs. This induces the activation of specific protein kinase complexes such as transforming growth factor--activated kinase 1 (TAK1), which, in turn, activate NF-B and MAPK signaling, leading to the expression of pro-inflammatory genes^[212]^.

Several studies clearly converged on the evidence that tumor-released EVs modulate the immune response via the TLR2-MyD88-NF-B pathway. Initially, Liu *et al*. reported the contribution of MyD88 in the differentiation of myeloid-derived suppressor cells by tumor cell-released EVs^[213]^, a finding confirmed by Chalmin *et al*.^[214]^. Interesting results were obtained by administering EVs from different body fluids and tumors (amniotic fluid, liver cirrhosis ascites and ovarian cancer ascites) to THP1 monocytes, as EV-mediated TLR-dependent signaling was reported to trigger NF-B activation, stimulating the production of pro-inflammatory cytokines^[215]^. Similarly, various types of macrophages were shown to internalize EVs, but only EVs isolated from cancer cells could stimulate TLR2 and MyD88-dependent activation of NF-B^[216]^. EVs also triggered a pro-inflammatory phenotype in MSCs, as EVs released by A549 lung cancer cells induced NF-B activation via TLR2^[217]^ [Figure 3B].

Some studies also investigated the effect of EVs on the activation of TLR4. In DCs, the expression of TLR4 was downregulated by pancreatic cancer cell-derived EVs^[218]^, whereas in neutrophils, the NF-B pathway was activated by gastric cancer cell-derived EVs via TLR4^[219]^. When non-polarized macrophages were co-cultured with EVs derived from a colorectal cancer and a multiple myeloma cell line, EVs induced pro-inflammatory cytokine expression via the TLR4 signaling pathway^[220]^. These studies confirmed the role of EVs released by cancer cells in creating pro-inflammatory conditions in the tumor microenvironment. Additionally, the direct transfer of TLR4 via EVs was also observed^[221]^: EVs released by DCs transfer TLR4 to DCs derived from TLR4-knockout mice, inducing the activation of NF-B signaling. This study clearly demonstrated that functional TLR receptors could be transferred, at least among DCs, via EVs.

Furthermore, studies on TLR activation by EVs revealed another important aspect, namely the activation of TLRs by the nucleic acid carried by EVs. Fabbri *et al*. reported that a few vesicular miRNAs (miR-21 and miR-29a) bound as ligands to human TLR8 (and murine TRL7), triggering an inflammatory response, possibly contributing to tumor growth and metastasis^[222]^. Liu *et al*. showed that lung epithelial cells were the target of vesicular RNAs via TLR3^[223]^. These EVs stimulated chemokine secretion by epithelial cells, promoting neutrophil recruitment and a lung pre-metastatic niche.

TLRs were not the only receptors targeted by EVs, as recent investigations showed that other PRRs could be modulated by EVs. The stimulator of interferon genes (STING) is part of a signaling pathway activated by cyclic GMP-AMP produced by cGAS upon stimulation by the presence of pathogenic DNA in the cytosol. STING activates the NF-B transcription factors and the expression of inflammatory cytokines. In the interplay between innate and adaptive immune response, the cGAS/STING cytosolic DNA-sensing pathway was stimulated in DCs by EVs released by activated T cells upon antigen contact, via their content in genomic and mitochondrial DNA, thus leading to antiviral responses^[224]^. EVs released by breast cancer cells treated with the chemotherapeutic topotecan also activated the STING signaling pathway in DCs via their DNA content, promoting anticancer immunity^[225]^. RLRs are mostly located in the cytosol and are activated by RNAs with specific biochemical features, such as viral RNAs with a triphosphate at their 5’ end. Activation of RLRs induces the transcription of type I interferons, known to play a pivotal role in the antiviral response. Studying the interaction between stromal fibroblasts and cancer cells, Nabet *et al*. discovered that in specific conditions, breast cancer cells released the RN7SL1 noncoding RNA without its binding proteins in EVs^[226]^. This “naked” RNA activated the RIG-I signaling pathway, promoting tumor growth and metastasis. Nucleotide-binding oligomerization domain-containing protein 1 (NOD1) recognizes bacterial peptidoglycan components, inducing inflammatory responses in macrophages by activation of NF-B and MAPK downstream pathways. EVs released from colorectal cancer cells prompted the secretion of inflammatory cytokines in macrophages in a NOD-1-dependent manner^[227]^. Altogether, these studies clearly indicate that EVs may be a vehicle for the horizontal dissemination of DAMP signals and play a role in activating cell signaling pathways that are important for the clearance of harmful stimuli via innate and adaptive immune responses^[187]^.

## CONCLUSION

EVs were initially identified as a tool for cell garbage disposal and later recognized as an additional means of cell-to-cell communication. The discovery of cell signaling molecules, both ligands and receptors, in EVs has also shed light on cell signaling pathways that had been investigated for years but whose biochemical mechanism of signal transmission had remained elusive. Wnt/β-catenin and Hh morphogenetic pathways both relied on membrane-anchored proteins released extracellularly, but it was not easy to figure out how they could reach their target, as they were not soluble. EVs have provided a solution to this riddle. Nevertheless, many issues still need to be addressed. For Wnt and Hh, alternative localization mechanisms beyond vesicular transport have been suggested, such as cell prolongations, specifically cytonemes. As for Notch and Eph/ephrin, EVs may represent an additional mechanism for activation of signals at a distance without cell-to-cell contact. Regarding TGF-β, a fraction of it is released via EVs and the rest as a soluble factor. There is evidence that soluble and EV-associated TGF-β may elicit responses of diverse intensity, higher for EV-associated TGF-β. Overall findings indicated that for these pathways, EV-mediated signaling co-exists with other mechanisms of signal transmission. As there are no means of blocking EV release, probably because different and simultaneous biogenetic pathways are present in the same cells, it is difficult to demonstrate to what extent these pathways depend on EV release. From an evolutionary point of view, the presence of morphogens such as Wnt and Hh in EVs poses the question of how ancient this mechanism of cell-to-cell communication is. Did Wnt and Hh evolve primarily using EVs as a tool to deliver signals or did this mode of transmission evolve later? How did Notch and Eph/ephrin begin to use EVs as a tool to transmit signals in addition to cell-to-cell contact? These issues are far from being elucidated. In addition, it is also worth mentioning that most information relies on studies investigating signaling abnormalities in cancer models. However, EV-associated signaling molecules also play relevant roles in other contexts, such as the differentiation and homeostasis of the nervous system. For this reason, the role of cell signaling modulation by EVs in neurodegenerative diseases may need to be specifically reviewed.

Technical matters are also relevant. In most articles published to date, cell and animal models were often subjected to repeated administration of high doses of EVs. Although results often indicated EV-mediated cell signaling and significant biological effects in target cells, more sophisticated experimental approaches, possibly employing more physiological EV concentrations and more sensitive detection techniques, are needed to demonstrate the role of EVs in mediating specific cell signaling *in vivo*, as well as the contribution of each specific EV subtype. Although separation methods for obtaining specific EV subtypes are still lacking, there is an indication that specific EV subtypes may be released upon the activation of specific pathways. Furthermore, stress conditions alter EV release, presumably favoring the release of some types of EVs at the expense of others. However, our current knowledge of the specific presence of cell signaling molecules on EV subpopulations upon specific stressing stimuli is also limited. Finally, studies based on the involvement of EVs in mediating gain or loss of function of other proteins, e.g. oncogenes, are mostly based on overexpression or inhibition of those candidates, and these perturbations may per se influence EV release. Therefore, caution is required when claiming that a specific factor alters the release and contents of EVs and their associated signaling properties. Recently, the evidence of a protein corona formation on the surface of EVs drew attention to the limitations of many studies, because older studies did not consider the presence of the EV corona, including in their experiments the degradation steps or other methods allowing the localization of bioactive molecules. In conclusion, current knowledge identifies EVs as playing a pivotal role in important and evolutionarily conserved signaling pathways, but a clearer picture will be obtained as advancements enable more efficient separation of EV subpopulations, shedding light on the heterogeneity and diversity of EVs in terms of signaling molecule contents and functional properties.

## DECLARATIONS

### Copyright

© The Author(s) 2024.

## References

[1] Couch Y, Buzàs EI, Di Vizio D (2021). A brief history of nearly EV-erything - the rise and rise of extracellular vesicles. J Extracell Vesicles.

[2] Vidal M (2019). Exosomes: revisiting their role as “garbage bags”. Traffic.

[3] Raposo G, Stahl PD (2019). Extracellular vesicles: a new communication paradigm?. Nat Rev Mol Cell Biol.

[4] Yáñez-Mó M, Siljander PR, Andreu Z (2015). Biological properties of extracellular vesicles and their physiological functions. J Extracell Vesicles.

[5] Kalluri R, LeBleu VS (2020). The biology, function, and biomedical applications of exosomes. Science.

[6] Clancy JW, Schmidtmann M, D'Souza-Schorey C (2021). The ins and outs of microvesicles. FASEB Bioadv.

[7] Battistelli M, Falcieri E (2020). Apoptotic bodies: particular extracellular vesicles involved in intercellular communication. Biology (Basel).

[8] Kowal J, Arras G, Colombo M (2016). Proteomic comparison defines novel markers to characterize heterogeneous populations of extracellular vesicle subtypes. Proc Natl Acad Sci U S A.

[9] Théry C, Witwer KW, Aikawa E (2018). Minimal information for studies of extracellular vesicles 2018 (MISEV2018): a position statement of the international society for extracellular vesicles and update of the MISEV2014 guidelines. J Extracell Vesicles.

[10] Welsh JA, Goberdhan DCI, O'Driscoll L, MISEV Consortium (2024). Minimal information for studies of extracellular vesicles (MISEV2023): from basic to advanced approaches. J Extracell Vesicles.

[11] Jiang Y, Liu X, Ye J (2023). Migrasomes, a new mode of intercellular communication. Cell Commun Signal.

[12] Ciardiello C, Migliorino R, Leone A, Budillon A (2020). Large extracellular vesicles: size matters in tumor progression. Cytokine Growth Factor Rev.

[13] Jeppesen DK, Zhang Q, Franklin JL, Coffey RJ (2023). Extracellular vesicles and nanoparticles: emerging complexities. Trends Cell Biol.

[14] Zhang H, Freitas D, Kim HS (2018). Identification of distinct nanoparticles and subsets of extracellular vesicles by asymmetric flow field-flow fractionation. Nat Cell Biol.

[15] Zhang Q, Jeppesen DK, Higginbotham JN (2021). Supermeres are functional extracellular nanoparticles replete with disease biomarkers and therapeutic targets. Nat Cell Biol.

[16] Colombo M, Moita C, van Niel G (2013). Analysis of ESCRT functions in exosome biogenesis, composition and secretion highlights the heterogeneity of extracellular vesicles. J Cell Sci.

[17] Urbanelli L, Magini A, Buratta S (2013). Signaling pathways in exosomes biogenesis, secretion and fate. Genes (Basel).

[18] (2018). Niel G, D'Angelo G, Raposo G. Shedding light on the cell biology of extracellular vesicles. Nat Rev Mol Cell Biol.

[19] Tricarico C, Clancy J, D'Souza-Schorey C (2017). Biology and biogenesis of shed microvesicles. Small GTPases.

[20] Sagini K, Costanzi E, Emiliani C, Buratta S, Urbanelli L (2018). Extracellular vesicles as conveyors of membrane-derived bioactive lipids in immune system. Int J Mol Sci.

[21] Hurley JH (2015). ESCRTs are everywhere. EMBO J.

[22] Chiaradia E, Tancini B, Emiliani C (2021). Extracellular vesicles under oxidative stress conditions: biological properties and physiological roles. Cells.

[23] Valadi H, Ekström K, Bossios A, Sjöstrand M, Lee JJ, Lötvall JO (2007). Exosome-mediated transfer of mRNAs and microRNAs is a novel mechanism of genetic exchange between cells. Nat Cell Biol.

[24] Li Z, Zhu X, Huang S (2020). Extracellular vesicle long non-coding RNAs and circular RNAs: biology, functions and applications in cancer. Cancer Lett.

[25] Liu DSK, Yang QZC, Asim M, Krell J, Frampton AE (2022). The clinical significance of transfer RNAs present in extracellular vesicles. Int J Mol Sci.

[26] Hur JY, Lee KY (2021). Characteristics and clinical application of extracellular vesicle-derived DNA. Cancers (Basel).

[27] Tóth EÁ, Turiák L, Visnovitz T (2021). Formation of a protein corona on the surface of extracellular vesicles in blood plasma. J Extracell Vesicles.

[28] Yates AG, Pink RC, Erdbrügger U (2022). In sickness and in health: the functional role of extracellular vesicles in physiology and pathology in vivo: part I: Health and Normal Physiology: Part I: Health and Normal Physiology. J Extracell Vesicles.

[29] Zabeo D, Cvjetkovic A, Lässer C, Schorb M, Lötvall J, Höög JL (2017). Exosomes purified from a single cell type have diverse morphology. J Extracell Vesicles.

[30] Prada I, Meldolesi J (2016). Binding and fusion of extracellular vesicles to the plasma membrane of their cell targets. Int J Mol Sci.

[31] Liu YJ, Wang C (2023). A review of the regulatory mechanisms of extracellular vesicles-mediated intercellular communication. Cell Commun Signal.

[32] French KC, Antonyak MA, Cerione RA (2017). Extracellular vesicle docking at the cellular port: extracellular vesicle binding and uptake. Semin Cell Dev Biol.

[33] O'Brien K, Ughetto S, Mahjoum S, Nair AV, Breakefield XO (2022). Uptake, functionality, and re-release of extracellular vesicle-encapsulated cargo. Cell Rep.

[34] Yoon YJ, Kim DK, Yoon CM (2014). Egr-1 activation by cancer-derived extracellular vesicles promotes endothelial cell migration via ERK1/2 and JNK signaling pathways. PLoS One.

[35] Zhou D, Zhai W, Zhang M (2018). Mesenchymal stem cell-derived extracellular vesicles promote apoptosis in RSC96 schwann cells through the activation of the ERK pathway. Int J Clin Exp Pathol.

[36] Zhang Y, Chen Y, Shi L (2022). Extracellular vesicles microRNA-592 of melanoma stem cells promotes metastasis through activation of MAPK/ERK signaling pathway by targeting PTPN7 in non-stemness melanoma cells. Cell Death Discov.

[37] Bai X, Zhang H, Li Z (2022). Platelet-derived extracellular vesicles encapsulate microRNA-34c-5p to ameliorate inflammatory response of coronary artery endothelial cells via PODXL-mediated P38 MAPK signaling pathway. Nutr Metab Cardiovasc Dis.

[38] Nakano M, Fujimiya M (2021). Potential effects of mesenchymal stem cell derived extracellular vesicles and exosomal miRNAs in neurological disorders. Neural Regen Res.

[39] Xin Q, Zhu W, He C, Liu T, Wang H (2023). The effect of different sources of mesenchymal stem cells on microglia states. Front Aging Neurosci.

[40] Adamo A, Brandi J, Caligola S (2019). Extracellular vesicles mediate mesenchymal stromal cell-dependent regulation of B cell PI3K-AKT signaling pathway and actin cytoskeleton. Front Immunol.

[41] Liu M, Qiu Y, Xue Z (2020). Small extracellular vesicles derived from embryonic stem cells restore ovarian function of premature ovarian failure through PI3K/AKT signaling pathway. Stem Cell Res Ther.

[42] Liu W, Yuan Y, Liu D (2021). Extracellular vesicles from adipose-derived stem cells promote diabetic wound healing via the PI3K-AKT-mTOR-HIF-1α signaling pathway. Tissue Eng Regen Med.

[43] Ma Y, Zhou D, Zhang H, Tang L, Qian F, Su J (2021). Human umbilical cord mesenchymal stem cell-derived extracellular vesicles promote the proliferation of schwann cells by regulating the PI3K/AKT signaling pathway via transferring miR-21. Stem Cells Int.

[44] Huang D, Rao D, Jin Q (2023). Role of CD147 in the development and diagnosis of hepatocellular carcinoma. Front Immunol.

[45] Wang SJ, Qiu ZZ, Chen FW (2022). Bone marrow mesenchymal stem cell-derived extracellular vesicles containing miR-181d protect rats against renal fibrosis by inhibiting KLF6 and the NF-κB signaling pathway. Cell Death Dis.

[46] Fafián-Labora JA, O'Loghlen A (2021). NF-κB/IKK activation by small extracellular vesicles within the SASP. Aging Cell.

[47] Wolpert L

[48] Matusek T, Marcetteau J, Thérond PP (2020). Functions of Wnt and Hedgehog-containing extracellular vesicles in development and disease. J Cell Sci.

[49] Nusse R, Varmus H (2012). Three decades of Wnts: a personal perspective on how a scientific field developed. EMBO J.

[50] Qin K, Yu M, Fan J (2024). Canonical and noncanonical Wnt signaling: multilayered mediators, signaling mechanisms and major signaling crosstalk. Genes Dis.

[51] Liu J, Xiao Q, Xiao J (2022). Wnt/β-catenin signalling: function, biological mechanisms, and therapeutic opportunities. Signal Transduct Target Ther.

[52] Daulat AM, Borg JP (2017). Wnt/planar cell polarity signaling: new opportunities for cancer treatment. Trends Cancer.

[53] Menck K, Heinrichs S, Baden C, Bleckmann A (2021). The WNT/ROR pathway in cancer: from signaling to therapeutic intervention. Cells.

[54] Chairoungdua A, Smith DL, Pochard P, Hull M, Caplan MJ (2010). Exosome release of β-catenin: a novel mechanism that antagonizes Wnt signaling. J Cell Biol.

[55] Gross JC, Chaudhary V, Bartscherer K, Boutros M (2012). Active Wnt proteins are secreted on exosomes. Nat Cell Biol.

[56] Koles K, Nunnari J, Korkut C (2012). Mechanism of evenness interrupted (Evi)-exosome release at synaptic boutons. J Biol Chem.

[57] Menck K, Klemm F, Gross JC, Pukrop T, Wenzel D, Binder C (2013). Induction and transport of Wnt 5a during macrophage-induced malignant invasion is mediated by two types of extracellular vesicles. Oncotarget.

[58] Dovrat S, Caspi M, Zilberberg A (2014). 14-3-3 and β-catenin are secreted on extracellular vesicles to activate the oncogenic Wnt pathway. Mol Oncol.

[59] Kalra H, Gangoda L, Fonseka P (2019). Extracellular vesicles containing oncogenic mutant β-catenin activate Wnt signalling pathway in the recipient cells. J Extracell Vesicles.

[60] Scavo MP, Depalo N, Rizzi F (2019). FZD10 carried by exosomes sustains cancer cell proliferation. Cells.

[61] Irmer B, Efing J, Reitnauer LE (2023). Extracellular vesicle-associated tyrosine kinase-like orphan receptors ROR1 and ROR2 promote breast cancer progression. Cell Commun Signal.

[62] Schubert A, Boutros M (2021). Extracellular vesicles and oncogenic signaling. Mol Oncol.

[63] Lin R, Wang S, Zhao RC (2013). Exosomes from human adipose-derived mesenchymal stem cells promote migration through Wnt signaling pathway in a breast cancer cell model. Mol Cell Biochem.

[64] Harada T, Yamamoto H, Kishida S (2017). Wnt5b-associated exosomes promote cancer cell migration and proliferation. Cancer Sci.

[65] Koch R, Demant M, Aung T (2014). Populational equilibrium through exosome-mediated Wnt signaling in tumor progression of diffuse large B-cell lymphoma. Blood.

[66] Chen Y, Zeng C, Zhan Y, Wang H, Jiang X, Li W (2017). Aberrant low expression of p85α in stromal fibroblasts promotes breast cancer cell metastasis through exosome-mediated paracrine Wnt10b. Oncogene.

[67] Qiu JJ, Sun SG, Tang XY, Lin YY, Hua KQ (2020). Extracellular vesicular Wnt7b mediates HPV E6-induced cervical cancer angiogenesis by activating the β-catenin signaling pathway. J Exp Clin Cancer Res.

[68] Hu YB, Yan C, Mu L (2019). Exosomal Wnt-induced dedifferentiation of colorectal cancer cells contributes to chemotherapy resistance. Oncogene.

[69] Lerner N, Schreiber-Avissar S, Beit-Yannai E (2020). Extracellular vesicle-mediated crosstalk between NPCE cells and TM cells result in modulation of Wnt signalling pathway and ECM remodelling. J Cell Mol Med.

[70] Kholia S, Herrera Sanchez MB, Deregibus MC, Sassoè-Pognetto M, Camussi G, Brizzi MF (2021). Human liver stem cell derived extracellular vesicles alleviate kidney fibrosis by interfering with the β-Catenin pathway through miR29b. Int J Mol Sci.

[71] Wang T, Zhang C, Meng X (2022). Long noncoding RNA metastasis-associated lung adenocarcinoma transcript 1 in extracellular vesicles promotes hepatic stellate cell activation, liver fibrosis and β-catenin signaling pathway. Front Physiol.

[72] Echelard Y, Epstein DJ, St-Jacques B (1993). Sonic Hedgehog, a member of a family of putative signaling molecules, is implicated in the regulation of CNS polarity. Cell.

[73] Jia Y, Wang Y, Xie J (2015). The Hedgehog pathway: role in cell differentiation, polarity and proliferation. Arch Toxicol.

[74] Doheny D, Manore SG, Wong GL, Lo HW (2020). Hedgehog signaling and truncated GLI1 in cancer. Cells.

[75] Kong JH, Siebold C, Rohatgi R (2019). Biochemical mechanisms of vertebrate Hedgehog signaling. Development.

[76] Liégeois S, Benedetto A, Garnier JM, Schwab Y, Labouesse M (2006). The V0-ATPase mediates apical secretion of exosomes containing Hedgehog-related proteins in caenorhabditis elegans. J Cell Biol.

[77] Matusek T, Wendler F, Polès S (2014). The ESCRT machinery regulates the secretion and long-range activity of Hedgehog. Nature.

[78] Gradilla AC, González E, Seijo I (2014). Exosomes as Hedgehog carriers in cytoneme-mediated transport and secretion. Nat Commun.

[79] Vyas N, Walvekar A, Tate D (2014). Vertebrate Hedgehog is secreted on two types of extracellular vesicles with different signaling properties. Sci Rep.

[80] Parchure A, Vyas N, Ferguson C, Parton RG, Mayor S (2015). Oligomerization and endocytosis of Hedgehog is necessary for its efficient exovesicular secretion. Mol Biol Cell.

[81] Coulter ME, Dorobantu CM, Lodewijk GA (2018). The ESCRT-III protein CHMP1A mediates secretion of sonic Hedgehog on a distinctive subtype of extracellular vesicles. Cell Rep.

[82] Hurbain I, Macé AS, Romao M (2022). Microvilli-derived extracellular vesicles carry Hedgehog morphogenic signals for drosophila wing imaginal disc development. Curr Biol.

[83] Zhao G, Li H, Guo Q (2020). Exosomal sonic Hedgehog derived from cancer-associated fibroblasts promotes proliferation and migration of esophageal squamous cell carcinoma. Cancer Med.

[84] Arasu UT, Deen AJ, Pasonen-Seppänen S (2020). HAS3-induced extracellular vesicles from melanoma cells stimulate IHH mediated c-Myc upregulation via the Hedgehog signaling pathway in target cells. Cell Mol Life Sci.

[85] Li L, Zhao J, Zhang Q (2021). Cancer cell-derived exosomes promote HCC tumorigenesis through Hedgehog pathway. Front Oncol.

[86] Bhat A, Yadav J, Thakur K (2021). Exosomes from cervical cancer cells facilitate pro-angiogenic endothelial reconditioning through transfer of Hedgehog-GLI signaling components. Cancer Cell Int.

[87] Zhou H, Li X, Wu RX (2021). Periodontitis-compromised dental pulp stem cells secrete extracellular vesicles carrying miRNA-378a promote local angiogenesis by targeting Sufu to activate the Hedgehog/Gli1 signalling. Cell Prolif.

[88] Ji Z, Cai Z, Gu S (2021). Exosomes derived from human adipose-derived stem cells inhibit lipogenesis involving Hedgehog signaling pathway. Front Bioeng Biotechnol.

[89] Sachan N, Sharma V, Mutsuddi M, Mukherjee A (2024). Notch signalling: multifaceted role in development and disease. FEBS J.

[90] Henrique D, Schweisguth F (2019). Mechanisms of Notch signaling: a simple logic deployed in time and space. Development.

[91] Steinbuck MP, Winandy S (2018). A review of Notch processing with new insights into ligand-independent Notch signaling in T-cells. Front Immunol.

[92] Zhou B, Lin W, Long Y (2022). Notch signaling pathway: architecture, disease, and therapeutics. Signal Transduct Target Ther.

[93] Ayaz F, Osborne BA (2014). Non-canonical notch signaling in cancer and immunity. Front Oncol.

[94] Xia R, Xu M, Yang J, Ma X (2022). The role of Hedgehog and Notch signaling pathway in cancer. Mol Biomed.

[95] Sheldon H, Heikamp E, Turley H (2010). New mechanism for Notch signaling to endothelium at a distance by Delta-like 4 incorporation into exosomes. Blood.

[96] Sharghi-Namini S, Tan E, Ong LL, Ge R, Asada HH (2014). Dll4-containing exosomes induce capillary sprout retraction in a 3D microenvironment. Sci Rep.

[97] Tan E, Asada HH, Ge R (2018). Extracellular vesicle-carried Jagged-1 inhibits HUVEC sprouting in a 3D microenvironment. Angiogenesis.

[98] Wang X, Jiao Y, Pan Y (2019). Fetal dermal mesenchymal stem cell-derived exosomes accelerate cutaneous wound healing by activating Notch signaling. Stem Cells Int.

[99] Wang Q, Lu Q (2017). Plasma membrane-derived extracellular microvesicles mediate non-canonical intercellular NOTCH signaling. Nat Commun.

[100] Boelens MC, Wu TJ, Nabet BY (2014). Exosome transfer from stromal to breast cancer cells regulates therapy resistance pathways. Cell.

[101] Wang B, Wang Y, Wang X (2022). Extracellular vesicles carrying miR-887-3p promote breast cancer cell drug resistance by targeting BTBD7 and activating the Notch1/Hes1 signaling pathway. Dis Markers.

[102] Yang J, Hu Y, Wang L, Sun X, Yu L, Guo W (2021). Human umbilical vein endothelial cells derived-exosomes promote osteosarcoma cell stemness by activating Notch signaling pathway. Bioengineered.

[103] Giannandrea D, Platonova N, Colombo M (2022). Extracellular vesicles mediate the communication between multiple myeloma and bone marrow microenvironment in a NOTCH dependent way. Haematologica.

[104] Pasquale EB (2010). Eph receptors and ephrins in cancer: bidirectional signalling and beyond. Nat Rev Cancer.

[105] Darling TK, Lamb TJ (2019). Emerging roles for eph receptors and ephrin ligands in immunity. Front Immunol.

[106] Zhao Y, Yin L, Zhang H, Lan T, Li S, Ma P (2018). Eph/ephrin family anchored on exosome facilitate communications between cells. Cell Biol Int.

[107] Choi DS, Park JO, Jang SC (2011). Proteomic analysis of microvesicles derived from human colorectal cancer ascites. Proteomics.

[108] Sun W, Zhao C, Li Y (2016). Osteoclast-derived microRNA-containing exosomes selectively inhibit osteoblast activity. Cell Discov.

[109] Gong J, Körner R, Gaitanos L, Klein R (2016). Exosomes mediate cell contact-independent ephrin-Eph signaling during axon guidance. J Cell Biol.

[110] Takasugi M, Okada R, Takahashi A, Virya Chen D, Watanabe S, Hara E (2017). Small extracellular vesicles secreted from senescent cells promote cancer cell proliferation through EphA2. Nat Commun.

[111] Sato S, Vasaikar S, Eskaros A (2019). EPHB2 carried on small extracellular vesicles induces tumor angiogenesis via activation of ephrin reverse signaling. JCI Insight.

[112] Han B, Zhang H, Tian R (2022). Exosomal EPHA2 derived from highly metastatic breast cancer cells promotes angiogenesis by activating the AMPK signaling pathway through Ephrin A1-EPHA2 forward signaling. Theranostics.

[113] Kany S, Vollrath JT, Relja B (2019). Cytokines in inflammatory disease. Int J Mol Sci.

[114] Rodrigues-Junior DM, Tsirigoti C, Lim SK, Heldin CH, Moustakas A (2022). Extracellular vesicles and transforming growth factor β signaling in cancer. Front Cell Dev Biol.

[115] Cohen MJ, Chirico WJ, Lipke PN (2020). Through the back door: unconventional protein secretion. Cell Surf.

[116] Frawley T, Piskareva O (2020). Extracellular vesicle dissemination of epidermal growth factor receptor and ligands and its role in cancer progression. Cancers (Basel).

[117] Morikawa M, Derynck R, Miyazono K (2016). TGF-β and the TGF-β family: context-dependent roles in cell and tissue physiology. Cold Spring Harb Perspect Biol.

[118] Robertson IB, Rifkin DB (2016). Regulation of the bioavailability of TGF-β and TGF-β-related proteins. Cold Spring Harb Perspect Biol.

[119] Chaikuad A, Bullock AN (2016). Structural basis of intracellular TGF-β signaling: receptors and Smads. Cold Spring Harb Perspect Biol.

[120] Zhang YE (2017). Non-Smad signaling pathways of the TGF-β family. Cold Spring Harb Perspect Biol.

[121] Xiang X, Poliakov A, Liu C (2009). Induction of myeloid-derived suppressor cells by tumor exosomes. Int J Cancer.

[122] Xie Y, Bai O, Yuan J (2009). Tumor apoptotic bodies inhibit CTL responses and antitumor immunity via membrane-bound transforming growth factor-beta1 inducing CD8^+^ T-cell anergy and CD4^+^ Tr1 cell responses. Cancer Res.

[123] Wada J, Onishi H, Suzuki H (2010). Surface-bound TGF-beta1 on effusion-derived exosomes participates in maintenance of number and suppressive function of regulatory T-cells in malignant effusions. Anticancer Res.

[124] Yamada N, Kuranaga Y, Kumazaki M, Shinohara H, Taniguchi K, Akao Y (2016). Colorectal cancer cell-derived extracellular vesicles induce phenotypic alteration of T cells into tumor-growth supporting cells with transforming growth factor-β1-mediated suppression. Oncotarget.

[125] Szczepanski MJ, Szajnik M, Welsh A, Whiteside TL, Boyiadzis M (2011). Blast-derived microvesicles in sera from patients with acute myeloid leukemia suppress natural killer cell function via membrane-associated transforming growth factor-beta1. Haematologica.

[126] Berchem G, Noman MZ, Bosseler M (2016). Hypoxic tumor-derived microvesicles negatively regulate NK cell function by a mechanism involving TGF-β and miR23a transfer. Oncoimmunology.

[127] Ludwig N, Yerneni SS, Azambuja JH (2022). TGFβ^+^ small extracellular vesicles from head and neck squamous cell carcinoma cells reprogram macrophages towards a pro-angiogenic phenotype. J Extracell Vesicles.

[128] Fu XH, Li JP, Li XY (2022). M2-macrophage-derived exosomes promote meningioma progression through TGF-β signaling pathway. J Immunol Res.

[129] Huang F, Wan J, Hu W, Hao S (2017). Enhancement of anti-leukemia immunity by leukemia-derived exosomes via downregulation of TGF-β1 expression. Cell Physiol Biochem.

[130] de Miguel-Perez D, Russo A, Gunasekaran M (2023). Baseline extracellular vesicle TGF-β is a predictive biomarker for response to immune checkpoint inhibitors and survival in non-small cell lung cancer. Cancer.

[131] Krishnamachary B, Mahajan A, Kumar A (2021). Extracellular vesicle TGF-β1 is linked to cardiopulmonary dysfunction in human immunodeficiency virus. Am J Respir Cell Mol Biol.

[132] Webber J, Steadman R, Mason MD, Tabi Z, Clayton A (2010). Cancer exosomes trigger fibroblast to myofibroblast differentiation. Cancer Res.

[133] Webber JP, Spary LK, Sanders AJ (2015). Differentiation of tumour-promoting stromal myofibroblasts by cancer exosomes. Oncogene.

[134] Shelke GV, Yin Y, Jang SC (2019). Endosomal signalling via exosome surface TGFβ-1. J Extracell Vesicles.

[135] (2018). Goulet C, Bernard G, Tremblay S, Chabaud S, Bolduc S, Pouliot F. Exosomes induce fibroblast differentiation into cancer-associated fibroblasts through TGFβ signaling. Mol Cancer Res.

[136] Gu J, Qian H, Shen L (2012). Gastric cancer exosomes trigger differentiation of umbilical cord derived mesenchymal stem cells to carcinoma-associated fibroblasts through TGF-β/Smad pathway. PLoS One.

[137] Baglio SR, Lagerweij T, Pérez-Lanzón M (2017). Blocking tumor-educated MSC paracrine activity halts osteosarcoma progression. Clin Cancer Res.

[138] Hao Y, Baker D, Ten Dijke P (2019). TGF-β-mediated epithelial-mesenchymal transition and cancer metastasis. Int J Mol Sci.

[139] Qu Z, Feng J, Pan H, Jiang Y, Duan Y, Fa Z (2019). Exosomes derived from HCC cells with different invasion characteristics mediated EMT through TGF-β/Smad signaling pathway. Onco Targets Ther.

[140] Nakayama F, Miyoshi M, Kimoto A (2022). Pancreatic cancer cell-derived exosomes induce epithelial-mesenchymal transition in human pancreatic cancer cells themselves partially via transforming growth factor β1. Med Mol Morphol.

[141] Yin Y, Shelke GV, Lässer C, Brismar H, Lötvall J (2020). Extracellular vesicles from mast cells induce mesenchymal transition in airway epithelial cells. Respir Res.

[142] Feng Y, Zhan F, Zhong Y, Tan B (2020). Effects of human umbilical cord mesenchymal stem cells derived from exosomes on migration ability of endometrial glandular epithelial cells. Mol Med Rep.

[143] Tan C, Sun W, Xu Z (2021). Small extracellular vesicles deliver TGF-β1 and promote adriamycin resistance in breast cancer cells. Mol Oncol.

[144] Borges FT, Melo SA, Özdemir BC (2013). TGF-β1-containing exosomes from injured epithelial cells activate fibroblasts to initiate tissue regenerative responses and fibrosis. J Am Soc Nephrol.

[145] Zhu QJ, Zhu M, Xu XX, Meng XM, Wu YG (2019). Exosomes from high glucose-treated macrophages activate glomerular mesangial cells via TGF-β1/Smad3 pathway *in vivo* and *in vitro*. FASEB J.

[146] Li J, Liu ZP, Xu C, Guo A (2020). TGF-β1-containing exosomes derived from bone marrow mesenchymal stem cells promote proliferation, migration and fibrotic activity in rotator cuff tenocytes. Regen Ther.

[147] Zhang L, Wei W, Ai X (2021). Extracellular vesicles from hypoxia-preconditioned microglia promote angiogenesis and repress apoptosis in stroke mice via the TGF-β/Smad2/3 pathway. Cell Death Dis.

[148] Han T, Song P, Wu Z (2022). MSC secreted extracellular vesicles carrying TGF-beta upregulate Smad 6 expression and promote the regrowth of neurons in spinal cord injured rats. Stem Cell Rev Rep.

[149] Languino LR, Singh A, Prisco M (2016). Exosome-mediated transfer from the tumour microenvironment increases TGFβ signaling in squamous cell carcinoma. Am J Transl Res.

[150] Gautheron F, Georgievski A, Garrido C, Quéré R (2023). Bone marrow-derived extracellular vesicles carry the TGF-β signal transducer Smad2 to preserve hematopoietic stem cells in mice. Cell Death Discov.

[151] Wee P, Wang Z (2017). Epidermal growth factor receptor cell proliferation signaling pathways. Cancers (Basel).

[152] Singh B, Carpenter G, Coffey RJ (2016). EGF receptor ligands: recent advances. F1000Res.

[153] Uribe ML, Marrocco I, Yarden Y (2021). EGFR in cancer: signaling mechanisms, drugs, and acquired resistance. Cancers (Basel).

[154] Al-Nedawi K, Meehan B, Micallef J (2008). Intercellular transfer of the oncogenic receptor EGFRvIII by microvesicles derived from tumour cells. Nat Cell Biol.

[155] Skog J, Würdinger T, van Rijn S (2008). Glioblastoma microvesicles transport RNA and proteins that promote tumour growth and provide diagnostic biomarkers. Nat Cell Biol.

[156] Sanderson MP, Keller S, Alonso A, Riedle S, Dempsey PJ, Altevogt P (2008). Generation of novel, secreted epidermal growth factor receptor (EGFR/ErbB1) isoforms via metalloprotease-dependent ectodomain shedding and exosome secretion. J Cell Biochem.

[157] Read J, Ingram A, Al Saleh HA (2017). Nuclear transportation of exogenous epidermal growth factor receptor and androgen receptor via extracellular vesicles. Eur J Cancer.

[158] Al-Nedawi K, Meehan B, Kerbel RS, Allison AC, Rak J (2009). Endothelial expression of autocrine VEGF upon the uptake of tumor-derived microvesicles containing oncogenic EGFR. Proc Natl Acad Sci U S A.

[159] Song X, Ding Y, Liu G (2016). Cancer cell-derived exosomes induce mitogen-activated protein kinase-dependent monocyte survival by transport of functional receptor tyrosine kinases. J Biol Chem.

[160] Gao L, Wang L, Dai T (2018). Tumor-derived exosomes antagonize innate antiviral immunity. Nat Immunol.

[161] Yu S, Sha H, Qin X (2020). EGFR E746-A750 deletion in lung cancer represses antitumor immunity through the exosome-mediated inhibition of dendritic cells. Oncogene.

[162] Fujiwara T, Eguchi T, Sogawa C (2018). Carcinogenic epithelial-mesenchymal transition initiated by oral cancer exosomes is inhibited by anti-EGFR antibody cetuximab. Oral Oncol.

[163] Zhang H, Deng T, Liu R (2017). Exosome-delivered EGFR regulates liver microenvironment to promote gastric cancer liver metastasis. Nat Commun.

[164] Meckes DG Jr, Shair KH, Marquitz AR, Kung CP, Edwards RH, Raab-Traub N (2010). Human tumor virus utilizes exosomes for intercellular communication. Proc Natl Acad Sci U S A.

[165] Higginbotham JN, Demory Beckler M, Gephart JD (2011). Amphiregulin exosomes increase cancer cell invasion. Curr Biol.

[166] Corrado C, Saieva L, Raimondo S, Santoro A, De Leo G, Alessandro R (2016). Chronic myelogenous leukaemia exosomes modulate bone marrow microenvironment through activation of epidermal growth factor receptor. J Cell Mol Med.

[167] Taverna S, Pucci M, Giallombardo M (2017). Amphiregulin contained in NSCLC-exosomes induces osteoclast differentiation through the activation of EGFR pathway. Sci Rep.

[168] Raimondo S, Saieva L, Vicario E (2019). Multiple myeloma-derived exosomes are enriched of amphiregulin (AREG) and activate the epidermal growth factor pathway in the bone microenvironment leading to osteoclastogenesis. J Hematol Oncol.

[169] Yang WW, Yang LQ, Zhao F (2017). Epiregulin promotes lung metastasis of salivary adenoid cystic carcinoma. Theranostics.

[170] Zhang Q, Higginbotham JN, Jeppesen DK (2019). Transfer of functional cargo in exomeres. Cell Rep.

[171] Shibuya M (2011). Vascular endothelial growth factor (VEGF) and its receptor (VEGFR) signaling in angiogenesis: a crucial target for anti- and pro-angiogenic therapies. Genes Cancer.

[172] Mou S, Zhou M, Li Y (2019). Extracellular vesicles from human adipose-derived stem cells for the improvement of angiogenesis and fat-grafting application. Plast Reconstr Surg.

[173] Masoumi-Dehghi S, Babashah S, Sadeghizadeh M (2020). microRNA-141-3p-containing small extracellular vesicles derived from epithelial ovarian cancer cells promote endothelial cell angiogenesis through activating the JAK/STAT3 and NF-κB signaling pathways. J Cell Commun Signal.

[174] Lee JK, Park SR, Jung BK (2013). Exosomes derived from mesenchymal stem cells suppress angiogenesis by down-regulating VEGF expression in breast cancer cells. PLoS One.

[175] Taraboletti G, D'Ascenzo S, Giusti I (2006). Bioavailability of VEGF in tumor-shed vesicles depends on vesicle burst induced by acidic pH. Neoplasia.

[176] Feng Q, Zhang C, Lum D (2017). A class of extracellular vesicles from breast cancer cells activates VEGF receptors and tumour angiogenesis. Nat Commun.

[177] Treps L, Perret R, Edmond S, Ricard D, Gavard J (2017). Glioblastoma stem-like cells secrete the pro-angiogenic VEGF-A factor in extracellular vesicles. J Extracell Vesicles.

[178] Wang CA, Chang IH, Hou PC (2020). DUSP2 regulates extracellular vesicle-VEGF-C secretion and pancreatic cancer early dissemination. J Extracell Vesicles.

[179] Wang CS, Kavalali ET, Monteggia LM (2022). BDNF signaling in context: from synaptic regulation to psychiatric disorders. Cell.

[180] Wang Y, Liang J, Xu B, Yang J, Wu Z, Cheng L (2024). TrkB/BDNF signaling pathway and its small molecular agonists in CNS injury. Life Sci.

[181] Barcellos N, Cechinel LR, de Meireles LCF (2020). Effects of exercise modalities on BDNF and IL-1β content in circulating total extracellular vesicles and particles obtained from aged rats. Exp Gerontol.

[182] Suire CN, Eitan E, Shaffer NC (2017). Walking speed decline in older adults is associated with elevated pro-BDNF in plasma extracellular vesicles. Exp Gerontol.

[183] Chung CC, Huang PH, Chan L, Chen JH, Chien LN, Hong CT (2020). Plasma exosomal brain-derived neurotrophic factor correlated with the postural instability and gait disturbance-related motor symptoms in patients with parkinson’s disease. Diagnostics (Basel).

[184] Solana-Balaguer J, Campoy-Campos G, Martín-Flores N (2023). Neuron-derived extracellular vesicles contain synaptic proteins, promote spine formation, activate TrkB-mediated signalling and preserve neuronal complexity. J Extracell Vesicles.

[185] Li D, Wu M (2021). Pattern recognition receptors in health and diseases. Signal Transduct Target Ther.

[186] Kawasaki T, Kawai T (2014). Toll-like receptor signaling pathways. Front Immunol.

[187] Picca A, Guerra F, Calvani R (2020). Extracellular vesicles and damage-associated molecular patterns: a pandora’s box in health and disease. Front Immunol.

[188] Fu M, Hu Y, Lan T, Guan KL, Luo T, Luo M (2022). The Hippo signalling pathway and its implications in human health and diseases. Signal Transduct Target Ther.

[189] Azad T, Rezaei R, Surendran A (2020). Hippo signaling pathway as a central mediator of receptors tyrosine kinases (RTKs) in tumorigenesis. Cancers (Basel).

[190] Misra JR, Irvine KD (2018). The Hippo signaling network and its biological functions. Annu Rev Genet.

[191] Hu J, Wang S, Xiong Z (2018). Exosomal Mst1 transfer from cardiac microvascular endothelial cells to cardiomyocytes deteriorates diabetic cardiomyopathy. Biochim Biophys Acta Mol Basis Dis.

[192] Ren Y, Wu Y, He W, Tian Y, Zhao X (2023). Exosomes secreted from bone marrow mesenchymal stem cells suppress cardiomyocyte hypertrophy through Hippo-YAP pathway in heart failure. Genet Mol Biol.

[193] Ji C, Zhang J, Zhu Y (2020). Exosomes derived from hucMSC attenuate renal fibrosis through CK1δ/β-TRCP-mediated YAP degradation. Cell Death Dis.

[194] Li Z, Zhang M, Zheng J (2021). Human umbilical cord mesenchymal stem cell-derived exosomes improve ovarian function and proliferation of premature ovarian insufficiency by regulating the Hippo signaling pathway. Front Endocrinol (Lausanne).

[195] Wang Y, Zhao M, Li W (2021). BMSC-derived small extracellular vesicles induce cartilage reconstruction of temporomandibular joint osteoarthritis via autotaxin-YAP Signaling axis. Front Cell Dev Biol.

[196] Sun H, Cao X, Gong A (2022). Extracellular vesicles derived from astrocytes facilitated neurite elongation by activating the Hippo pathway. Exp Cell Res.

[197] Li FL, Guan KL (2022). The two sides of Hippo pathway in cancer. Semin Cancer Biol.

[198] Zanconato F, Cordenonsi M, Piccolo S (2016). YAP/TAZ at the roots of cancer. Cancer Cell.

[199] Furth N, Aylon Y (2017). The LATS1 and LATS2 tumor suppressors: beyond the Hippo pathway. Cell Death Differ.

[200] Song H, Mak KK, Topol L (2010). Mammalian Mst1 and Mst2 kinases play essential roles in organ size control and tumor suppression. Proc Natl Acad Sci U S A.

[201] Wang Z, Yuan Y, Ji X (2021). The Hippo-TAZ axis mediates vascular endothelial growth factor C in glioblastoma-derived exosomes to promote angiogenesis. Cancer Lett.

[202] Wang S, Su X, Xu M (2019). Exosomes secreted by mesenchymal stromal/stem cell-derived adipocytes promote breast cancer cell growth via activation of Hippo signaling pathway. Stem Cell Res Ther.

[203] Wang W, Wu L, Tian J (2022). Cervical cancer cells-derived extracellular vesicles containing microRNA-146a-5p affect actin dynamics to promote cervical cancer metastasis by activating the Hippo-YAP signaling pathway via WWC2. J Oncol.

[204] Yang P, Zhang D, Wang T (2022). CAF-derived exosomal WEE2-AS1 facilitates colorectal cancer progression via promoting degradation of MOB1A to inhibit the Hippo pathway. Cell Death Dis.

[205] Wang H, Min J, Xu C

[206] Saxena M, Yeretssian G (2014). NOD-like receptors: master regulators of inflammation and cancer. Front Immunol.

[207] Rehwinkel J, Gack MU (2020). RIG-I-like receptors: their regulation and roles in RNA sensing. Nat Rev Immunol.

[208] Motwani M, Pesiridis S, Fitzgerald KA (2019). DNA sensing by the cGAS-STING pathway in health and disease. Nat Rev Genet.

[209] Drouin M, Saenz J, Chiffoleau E (2020). C-Type lectin-like receptors: head or tail in cell death immunity. Front Immunol.

[210] Duan T, Du Y, Xing C, Wang HY, Wang RF (2022). Toll-like receptor signaling and its role in cell-mediated immunity. Front Immunol.

[211] Mielcarska MB, Bossowska-Nowicka M, Toka FN (2020). Cell surface expression of endosomal toll-like receptors-a necessity or a superfluous duplication?. Front Immunol.

[212] Balka KR, De Nardo D (2019). Understanding early TLR signaling through the myddosome. J Leukoc Biol.

[213] Liu Y, Xiang X, Zhuang X (2010). Contribution of MyD88 to the tumor exosome-mediated induction of myeloid derived suppressor cells. Am J Pathol.

[214] Chalmin F, Ladoire S, Mignot G (2010). Membrane-associated Hsp72 from tumor-derived exosomes mediates STAT3-dependent immunosuppressive function of mouse and human myeloid-derived suppressor cells. J Clin Invest.

[215] Bretz NP, Ridinger J, Rupp AK (2013). Body fluid exosomes promote secretion of inflammatory cytokines in monocytic cells via Toll-like receptor signaling. J Biol Chem.

[216] Chow A, Zhou W, Liu L (2014). Macrophage immunomodulation by breast cancer-derived exosomes requires Toll-like receptor 2-mediated activation of NF-κB. Sci Rep.

[217] Li X, Wang S, Zhu R, Li H, Han Q, Zhao RC (2016). Lung tumor exosomes induce a pro-inflammatory phenotype in mesenchymal stem cells via NFκB-TLR signaling pathway. J Hematol Oncol.

[218] Ding G, Zhou L, Qian Y (2015). Pancreatic cancer-derived exosomes transfer miRNAs to dendritic cells and inhibit RFXAP expression via miR-212-3p. Oncotarget.

[219] Zhang X, Shi H, Yuan X, Jiang P, Qian H, Xu W (2018). Tumor-derived exosomes induce N2 polarization of neutrophils to promote gastric cancer cell migration. Mol Cancer.

[220] Pucci M, Raimondo S, Urzì O (2021). Tumor-derived small extracellular vesicles induce pro-inflammatory cytokine expression and PD-L1 regulation in M0 macrophages via IL-6/STAT3 and TLR4 signaling pathways. Int J Mol Sci.

[221] Zhang Y, Meng J, Zhang L, Ramkrishnan S, Roy S (2019). Extracellular vesicles with exosome-like features transfer TLRs between dendritic cells. Immunohorizons.

[222] Fabbri M, Paone A, Calore F (2012). MicroRNAs bind to toll-like receptors to induce prometastatic inflammatory response. Proc Natl Acad Sci U S A.

[223] Liu Y, Gu Y, Han Y (2016). Tumor exosomal RNAs promote lung pre-metastatic niche formation by activating alveolar epithelial TLR3 to recruit neutrophils. Cancer Cell.

[224] Torralba D, Baixauli F, Villarroya-Beltri C (2018). Priming of dendritic cells by DNA-containing extracellular vesicles from activated T cells through antigen-driven contacts. Nat Commun.

[225] Kitai Y, Kawasaki T, Sueyoshi T (2017). DNA-containing exosomes derived from cancer cells treated with topotecan activate a STING-dependent pathway and reinforce antitumor immunity. J Immunol.

[226] Nabet BY, Qiu Y, Shabason JE (2017). Exosome RNA unshielding couples stromal activation to pattern recognition receptor signaling in cancer. Cell.

[227] Wei X, Ye J, Pei Y (2022). Extracellular vesicles from colorectal cancer cells promote metastasis via the NOD1 signalling pathway. J Extracell Vesicles.

